# Hyd ubiquitinates the NF-κB co-factor Akirin to operate an effective immune response in *Drosophila*

**DOI:** 10.1371/journal.ppat.1008458

**Published:** 2020-04-27

**Authors:** Alexandre Cammarata-Mouchtouris, Xuan-Hung Nguyen, Adrian Acker, François Bonnay, Akira Goto, Amir Orian, Marie-Odile Fauvarque, Michael Boutros, Jean-Marc Reichhart, Nicolas Matt

**Affiliations:** 1 Université de Strasbourg, CNRS, M3I UPR 9022, Strasbourg, France; 2 Vinmec Research Institute of Stem Cell and Gene Technology (VRISG) and College of Health Sciences, VinUniversity Hanoi, Vietnam; 3 Institute of Molecular Biotechnology of the Austrian Academy of Sciences (IMBA), Vienna, Austria; 4 Rappaport Research Institute and Rappaport Faculty of Medicine, Technion Integrated Cancer Center, Technion—Israel Institute of Technology, Haifa, Israel; 5 Université Grenoble Alpes, CEA, Inserm, BGE U1038, Grenoble, France; 6 Division of Signaling and Functional Genomics, German Cancer Research Center (DKFZ), and Department for Cell and Molecular Biology, Medical Faculty Mannheim, Heidelberg University, Heidelberg, Germany; University of Oxford, UNITED KINGDOM

## Abstract

The Immune Deficiency (IMD) pathway in *Drosophila melanogaster* is activated upon microbial challenge with Gram-negative bacteria to trigger the innate immune response. In order to decipher this nuclear factor κB (NF-κB) signaling pathway, we undertook an *in vitro* RNAi screen targeting E3 ubiquitin ligases specifically and identified the HECT-type E3 ubiquitin ligase Hyperplastic discs (Hyd) as a new actor in the IMD pathway. Hyd mediated Lys^63^ (K63)-linked polyubiquitination of the NF-κB cofactor Akirin was required for efficient binding of Akirin to the NF-κB transcription factor Relish. We showed that this Hyd-dependent interaction was required for the transcription of immunity-related genes that are activated by both Relish and Akirin but was dispensable for the transcription of genes that depend solely on Relish. Therefore Hyd is key in NF-κB transcriptional selectivity downstream of the IMD pathway. *Drosophila* depleted of Akirin or Hyd failed to express the full set of genes encoding immune-induced anti-microbial peptides and succumbed to immune challenges. We showed further that UBR5, the mammalian homolog of Hyd, was also required downstream of the NF-κB pathway for the activation of *Interleukin 6* (*IL6*) transcription by LPS or IL-1β in cultured human cells. Our findings link the action of an E3 ubiquitin ligase to the activation of immune effector genes, deepening our understanding of the involvement of ubiquitination in inflammation and identifying a potential target for the control of inflammatory diseases.

## Author summary

Ubiquitination has been recently identified in pathogenesis and progression of various diseases where inflammation is critical. NF-κB transcription factors are key actors in the transcriptional cascade leading to inflammation as they activate genes with pro- or anti-inflammatory activities. The similarity between the immune pathways in flies and mammals makes *Drosophila melanogaster* an excellent model to study the innate response. Accordingly, we decided to identify E3 ubiquitin-ligases involved in the regulation of NF-κB pathway, using *Drosophila* as a model system. A RNAi based screen in immortalized embryonic macrophage-like *Drosophila* cells points to the HECT-E3 ubiquitin ligase Hyd as a new regulator of the Immune-deficiency (IMD) NF-κB pathway, activated after Gram-negative immune challenge.

More precisely, we showed that Hyd acts at the level of Akirin, an evolutionarily conserved player in the NF-κB pathway, required for the transcription of pro-inflammatory genes, but not for the NF-κB-dependent genes contributing to the down-regulation of inflammation.

In addition, we could show that the human homologue of Hyd (UBR5) acts genetically at the level of human AKIRIN2, pointing to a unique dichotomy between Hyd/Akirin-dependent and -independent gene activation, allowing for the decoupling activation and resolution of inflammation. These results identified UBR5 as a putative target for anti-inflammatory compounds.

## Introduction

During evolution, metazoans developed strategies to effectively protect themselves from microbial threats. Since the molecular pathways mediating the innate immune response in insects and mammals are conserved, the fruit fly *Drosophila melanogaster* is a relevant model to explore the immune response [1, 2]. In *Drosophila* [3], the defense against microbes is executed mainly through the production of antimicrobial peptides (AMPs) under the control of two NF-κB transcription factors: Dorsal-related Immunity Factor (DIF) and Relish, respectively acting downstream of Toll and IMD pathways and homologues of mammalian RelB and p50 transcription factors.

Posttranslational regulation of proteins by the ubiquitin pathway is key for proper immune response [4]. The conjugation of ubiquitin polymers to target proteins by an ubiquitin ligase is a key mechanism for controlling the activity, localization, or stability of the targets. Lysine (Lys) residues of proteins can be modified by a polymer of ubiquitin (polyubiquitin) linked through Lys^48^ (K48) or Lys^63^ (K63) of ubiquitin molecules. Whereas K48-linked polyubiquitin mainly triggers degradation of proteins by the proteasome, K63-linked polyubiquitin mainly regulates the activity and the subcellular localization of proteins by modifying their protein-protein interactions [5]. In both mammals and *Drosophila*, ubiquitination is involved at various levels of the NF-κB pathways [6]. Furthermore, deregulation of ubiquitin ligases is implicated in inflammatory pathologies [7, 8] and tumor progression [9]. HECT-domain E3 ligases directly attach ubiquitin to a substrate, conversely to RING domain E3 ubiquitin ligases [10]. To achieve selectivity and specificity toward their substrates, HECT ubiquitin E3 ligases are tightly regulated, thus the identification of their substrates and regulators is critical in developing targets for drug discovery in the treatment of HECT E3 ubiquitin ligases related diseases [11, 12].

In *Drosophila*, Death-associated inhibitor of apoptosis 2 (Diap2) is the only E3 ubiquitin ligase identified thus far as a positive regulator of the IMD pathway [13, 14]. IMD protein functions as an adaptor protein in the IMD signaling pathway. The activation of pathogen recognition receptors (PRRs) by bacterial infection initiates the IMD cascade that transduces immunity signals notably through Diap2 [15, 16]. This protein is involved in the formation and activation of protein complexes organized around the IMD protein. To deepen our understanding of NF-κB pathway regulation by the ubiquitin system, we focused on identifying *Drosophila* ubiquitin ligases that are required for the activity of the IMD pathway through a RNAi-based screen in cultured *Drosophila* S2 cells.

Several E3 ubiquitin ligases emerged from our screen as positive or negative regulators of the IMD pathway. We focused on Hyperplastic discs (Hyd) because it was the only HECT-type E3 ubiquitin ligase identified in this screen and it had also emerged as a potential IMD pathway regulator in a parallel pilot screen [17]. Our results showed that Hyd was required *in vivo* for flies to survive an immune challenge with Gram-negative bacteria. Genetic epistasis analysis revealed that Hyd acted at the level of the NF-κB co-factor Akirin, which orchestrates the activation of a subset of NF-κB target genes in combination with the SWI/SNF chromatin remodeling complex [18–20].

We showed that Hyd decorated Akirin with K63-polyubiquitin chains that were required for Akirin binding to the NF-κB homolog Relish. Furthermore, we observed that the human ortholog of Hyd, UBR5 (also known as EDD1) [21], played a conserved role in NF-κB signaling in human cell lines. Similarly to human-AKIRIN2 (AKIRIN2), UBR5 was required for the activation of only a subset of NF-κB target genes after stimulation by LPS or IL-1β in cultured human cells. Thus, upon immune challenge, ubiquitin chains are instrumental to bridge NF-κB and its co-factor Akirin to activate an effective immune response.

## Results

### The E3 ubiquitin ligase Hyd is required for activation of the IMD pathway

To identify E3 ubiquitin ligases that modulate the IMD pathway, we screened a library of 174 double-strand RNAs (dsRNAs) targeting *Drosophila* proteins classified as putative E3 ubiquitin ligases in Flybase [22]. We used stably transfected *Drosophila* S2 cells expressing the *Attacin-A-luciferase* gene, a reporter of activation of the IMD pathway upon immune challenge with Gram-negative bacteria [23]. We evaluated the ability of dsRNA targeting each putative E3 ubiquitin ligase to interfere with the luciferase reporter upon stimulation of cells with heat-killed *Escherichia coli* (HKE), a general IMD pathway agonist (S1 Table).

**Fig 1:**
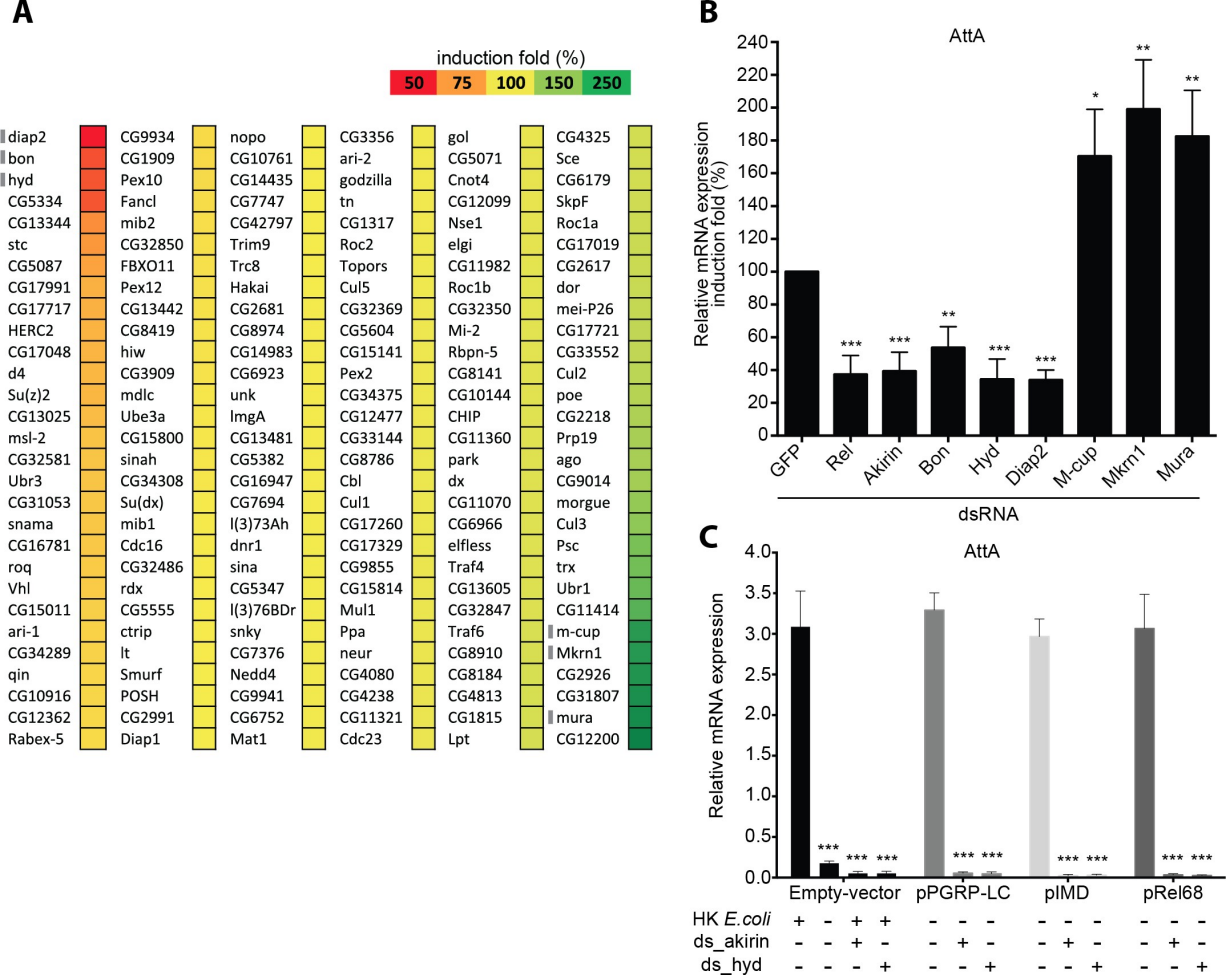
E3-ubiquitin ligases screen identified *in vitro* Hyd as involved in IMD pathway. (**A**) Induction of the IMD pathway measured by luciferase activity. 174 *Drosophila* E3 ubiquitin ligases were knocked down in S2 cells harboring the *Attacin-A–luciferase* reporter gene. Cells were transfected with individual dsRNAs targeting each E3 ligase before the IMD pathway was induced by stimulating the cells with heat-killed *E*. *coli* (HKE). Luciferase activity is expressed as an induction percentage compared to control cells treated with dsRNA targeting *GFP*. Three independent experiments were performed. Genes indicated by a gray bar were analyzed in Fig 1B. (**B**) Quantitative RT-PCR of *Attacin-A* mRNA from HKE-stimulated S2 cells transfected with dsRNA targeting *GFP* (negative control), *Relish* or *Akirin* (positive controls), and a subset of E3 ubiquitin ligases. After the ratio of stimulated over unstimulated values for each condition was determined, statistical significance was calculated by comparing genes knockdown to *GFP* dsRNA control. (**C**) Genetic epistasis experiment to place Hyd within the IMD pathway in S2 cells. The IMD pathway was induced by HKE stimulation or by transfecting the cells with *Pgrp-LC*, *Imd-V5*, or *Rel-HA* expressing plasmids. Cells were also transfected with dsRNA targeting *Akirin* or *Hyd*. Statistical significance was established by comparing values from the different conditions with cells treated with empty vector alone. Data are represented as mean ± standard deviation of three independent experiments (B-C). *P-value < 0.05; **P-value < 0.01; ***P-value < 0.001.

Diap2 is an E3 ubiquitin ligase that positively regulates the pathway by binding, polyubiquitinating and activating Death-related ced-3/Nedd2-like caspase (Dredd) which is required for Relish-mediated induction of antimicrobial peptides [24, 25]. Knockdown of *Diap2* resulted in a strong decrease of the luciferase reporter induction upon immune stimulation relative to the control *GFP*-targeting dsRNA (*dsGFP*) that does not target any transcripts present in the cells (Fig 1A), providing proof of concept for the screen. Knockdown of six E3-ubiquitin ligases (*M-cup*, *Mkrn1*, *CG2926*, *CG31807*, *Mura*, and *CG12200*) resulted in a strong increase in reporter activity upon immune stimulation. Therefore these E3 ubiquitin ligases behave as negative regulators of the IMD pathway. Conversely, knockdown of two Really Interesting New Gene (RING) domain E3-ubiquitin ligases, *Bon* and *CG5334*, or of the homologous to the E6-AP carboxyl terminus (HECT) domain E3 ubiquitin ligase *Hyd* resulted in an important decrease in reporter activity (Fig 1A). This suggests that Bon, CG5334, and Hyd are positive regulators of the IMD pathway. We decided to focus on Hyd because it was the only HECT domain E3-ubiquitin ligase we identified.

To validate the reporter assay, we transfected *Drosophila* S2 cells with dsRNA targeting either the NF-κB factor *Relish*, the Relish cofactor *Akirin*, *Hyd*, or E3 ubiquitin ligases chosen randomly among those that presented in the screen the strongest increase or decrease of *Attacin-A* induction (*Bon*, *Diap2*, *M-cup*, *Mkrn1* and *Mura*). Then, we stimulated the cells with HKE and monitored endogenous *Attacin-A* mRNA by quantitative reverse transcription PCR (RT-qPCR). Reducing the abundance of Relish, Akirin, or Hyd significantly decreased HKE-mediated *Attacin-A* induction, compared to cells treated with *dsGFP* (Fig 1B and S1 Fig). We observed that the RING-domain E3 ubiquitin ligases Bon, M-cup, Mkrn1, and Mura were required for the normal activation of *Attacin-A* expression and that the HECT E3 ubiquitin ligase Hyd acted as a positive regulator of *Attacin-A* expression in *Drosophila* S2 cells (Fig 1B).

In order to identify at which level of the IMD pathway Hyd is required, we undertook an epistasis analysis. *Drosophila* S2 cells were treated by dsRNA targeting *Hyd* or *Akirin* as a control and the IMD pathway was activated at different levels by transfecting either a truncated form of *PeptidoGlycan Receptor Protein-Long Chain a* (*Pgrp-LCa*), *Imd* or the 68kD active-form of Relish (*Rel68*) [18]. Measurement of *Attacin-A* expression by RT-qPCR assessed activation of the IMD pathway. We show that Hyd was required at the same level of Relish (Fig 1C) to exert its positive regulation on IMD pathway activation.

### Hyd acts at the level of Akirin to trigger full activation of the IMD pathway

**Fig 2:**
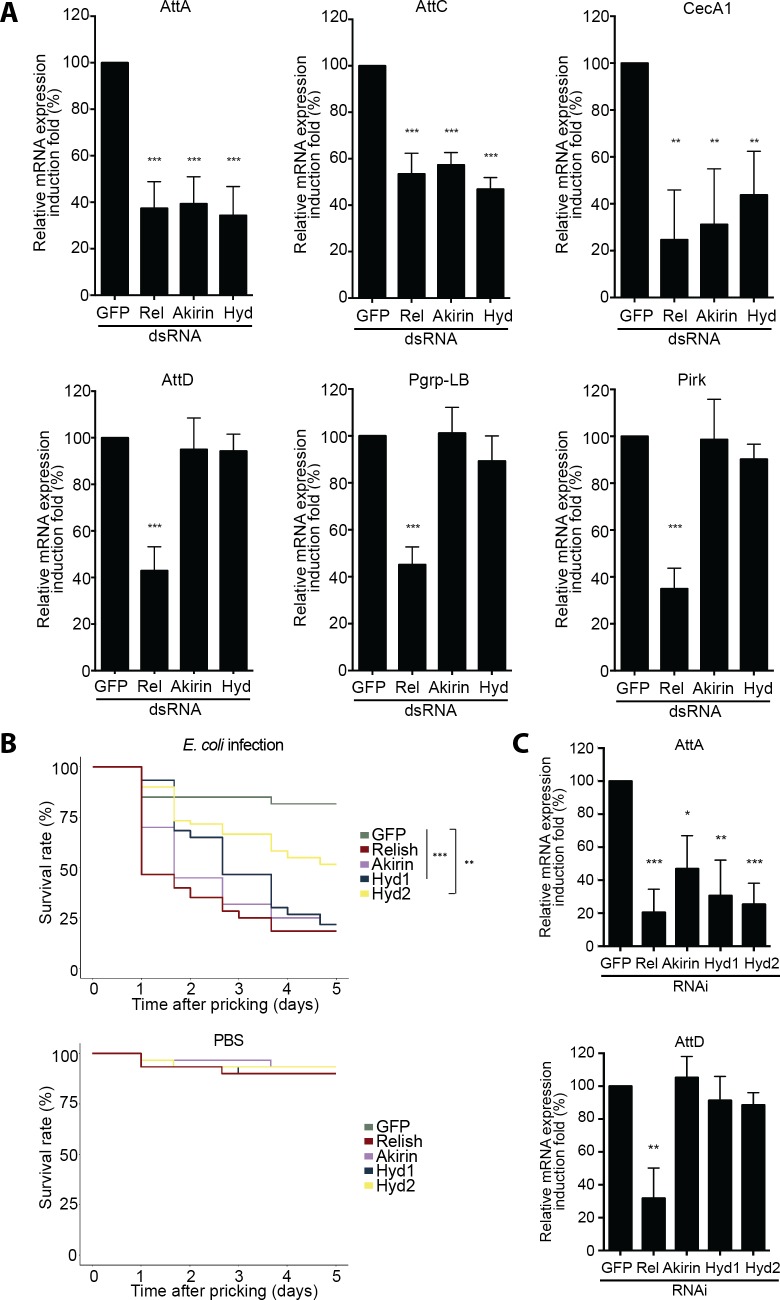
Hyd is required for the full activation of IMD response. (**A**) Quantitative RT-PCR of *Attacin-A*, *Attacin-C*, *Cecropin-A1*, *Attacin-D*, *Pgrp-LB* and *Pirk* mRNA from HKE-stimulated S2 cells transfected with dsRNA targeting *GFP* (negative control), *Relish* or *Akirin* (positive controls), and *Hyd*. (**B**) *In vivo* survival experiments performed on *C564-Gal4/UAS-RNAi* females *Drosophila*. They were infected with *E*. *coli* by septic injury (with PBS pricking as control). (**C**) Quantitative RT-PCR of *Attacin-A* and *Attacin-D* mRNA from *C564-Gal4/UAS-RNAi Drosophila* males infected with *E*. *coli* by septic injury. Data are represented as mean ± standard deviation of three independent experiments (A-C). After the ratio of stimulated over unstimulated values for each condition was determined, statistical significance was established by comparing genes knockdown with *GFP* dsRNA or RNAi control (B-C). *P-value < 0.05; **P-value < 0.01; ***P-value < 0.001.

Downstream of the IMD pathway, Relish target genes are divided into two subsets: genes that depend only on Relish for their expression (including *Attacin-D*, *Pgrp-LB*, *Pirk* and the majority of negative regulators) and ones requiring Akirin, and the deposition of an acetyl group on the lysine 4 of the histone 3 (H3K4ac), in addition to Relish (including *Attacin-A*, *Attacin-C*, *CecA1* and the majority of effectors) [19]. Upon immune challenge in S2 cells, using RT-qPCR, we observed that Hyd depletion recapitulated the immune phenotype of cells depleted for Akirin (Fig 2A). Consequently, Hyd is acting on Akirin-dependent NF-κB transcriptional selectivity *in vitro*.

We next investigated if Akirin and Hyd were similarly required for NF-κB transcriptional selectivity *in vivo*, using RNAi. As *Drosophila* embryonic development is impaired in absence of Akirin, we used the *C564-Gal4* transgene [26] to express RNAi constructs targeting *Akirin*, *Hyd* and *Relish* in the adult fat body, the main immune organ of *Drosophila [3]*. Flies depleted of Akirin (*C564-Gal4/UAS-RNAi-akirin*), Relish (*C564-Gal4/UAS-RNAi-relish*) or Hyd (*C564-Gal4/UAS-RNAi-hyd1* or *C564-Gal4/UAS-RNAi-hyd2*) displayed an impaired survival following *E*. *coli* infection when compared to control flies (*C564-Gal4/UAS-RNAi-GFP*) or to PBS pricking (Fig 2B and S2 Fig).

Following immune challenge by *E*. *coli*, expression of *Attacin-A*, but not of *Attacin-D*, was reduced in the absence of Akirin or Hyd, when compared to control flies (*C564-Gal4/UAS-RNAi-GFP*) (Fig 2C).

Our results suggest that Hyd is required at the level of Relish to activate the Akirin-dependent subset of Relish target genes during the immune response, allowing *Drosophila* to survive a Gram-negative bacterial challenge.

### Hyd-mediated K63-polyubiquitination of Akirin is critical for Akirin binding to Relish

**Fig 3:**
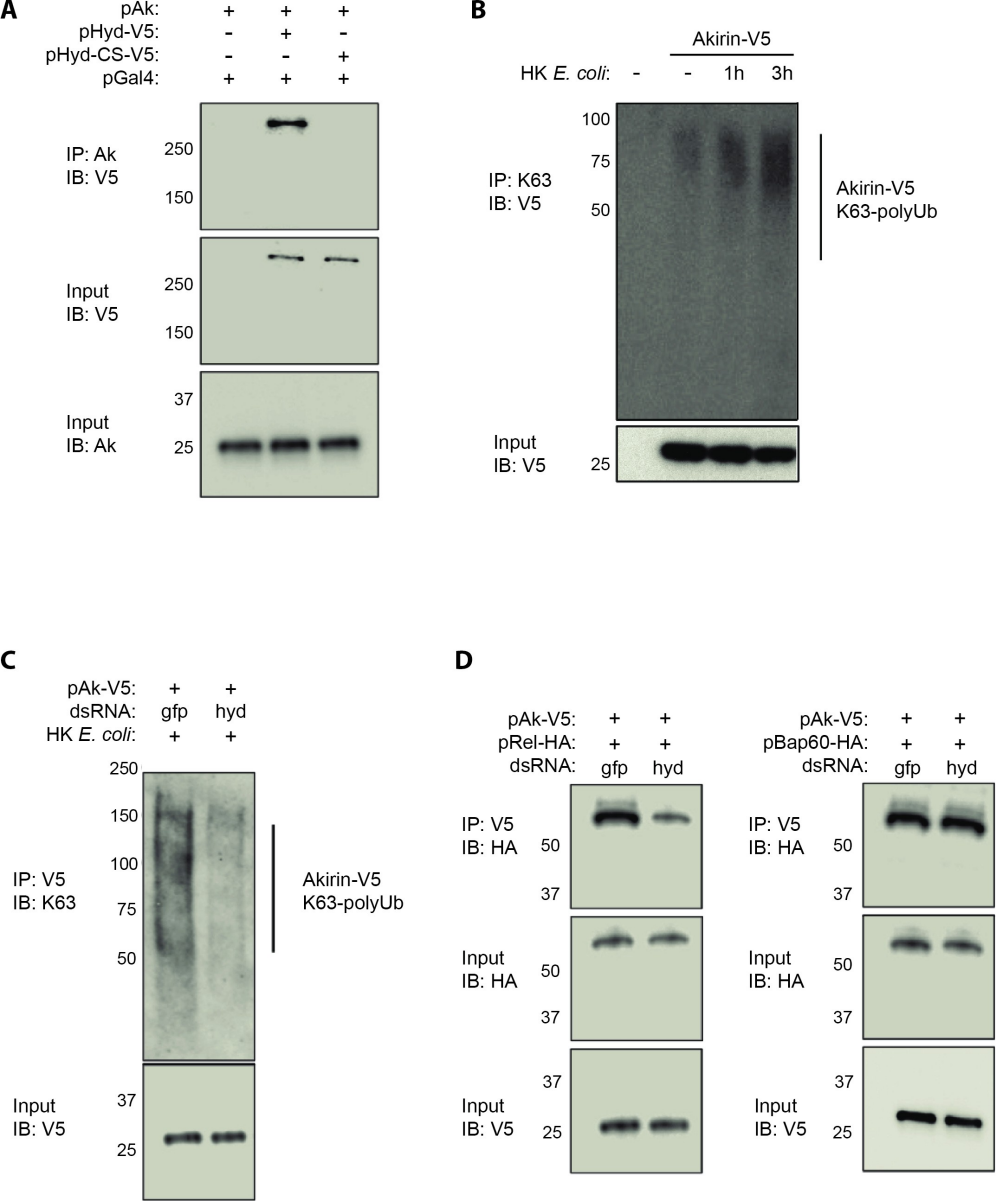
Hyd mediated-ubiquitination of Akirin is necessary for interaction with Relish. (**A**) Co-immunoprecipitation assay between over-expressed *Akirin* and *Hyd* in S2 cells. The cells were transiently transfected with *Akirin*, *Gal4*, *Hyd-V5* and/or *Hyd-CS-V5* expressing plasmids. Cell lysates were immunoprecipitated with anti-Akirin coupled agarose beads. Immunoprecipitates were analyzed by Western blotting with anti-V5 or anti-Akirin antibodies. (**B**) Immunoprecipitation assay of K63-polyUb chains on Akirin before and after immune challenge. S2 cells were transiently transfected with *Akirin-V5* expressing plasmid. Cell lysates were immunoprecipitated with anti-K63-polyUb coupled agarose beads. Immunoprecipitates were analyzed by Western blotting with anti-V5 antibodies. (**C**) Immunoprecipitation assay of Akirin after immune challenge. S2 cells were transiently transfected with *Akirin-V5* expressing plasmid and dsRNA targeting *GFP* or *Hyd*. Cell lysates were immunoprecipitated with anti-V5 coupled agarose beads. Immunoprecipitates were analyzed by Western blotting with anti-K63-polyUb and anti-V5 antibodies. (**D**) Co-immunoprecipitation assay between over-expressed *Akirin* and *Relish* or *Bap60* in S2 cells. The cells were transiently transfected with *Akirin-V5* and *Rel-HA* or *Bap60-HA* expressing plasmids and dsRNA targeting *GFP* or *Hyd*. Cell lysates were immunoprecipitated with anti-V5 coupled agarose beads. Immunoprecipitates were analyzed by Western blotting with anti-HA or anti-V5 antibodies. Data are representative of 2 independent experiments.

We next investigated if Akirin could be a *bona fide* target for the E3 ubiquitin-ligase Hyd. Co-immunoprecipitation assay in S2 cells showed that V5-tagged Hyd (Hyd-V5) [27] binds to endogenous Akirin (Fig 3A). By contrast, V5-tagged Hyd-CS (Hyd-CS-V5), which displays a mutated HECT domain by conversion of the catalytic cysteine at position 2854 to serine [27], is unable to bind to Akirin (Fig 3A). As a control we confirmed that Diap2, the E3-ubiquitin ligase acting upstream of Akirin in the IMD signaling cascade [13, 14], does not interact with Akirin (S3 Fig).

Protein extracts from cells transfected with a tagged version of Akirin (Akirin-V5) were immunoprecipitated with an anti-V5 antibody. Using cellular fractioning, we showed that upon immune challenge, polyubiquitinated Akirin-V5 accumulated in the nucleus of *Drosophila* S2 cells (S4 Fig). Western-blot experiments with antibodies targeting K63-polyUb chains showed that Akirin was K63-polyubiquitinated 1h and 3h after immune challenge with HKE (Fig 3B). This immune-induced post-translational modification of Akirin was attenuated upon knockdown of *Hyd* (Fig 3C).

According to literature, Hyd is suspected to also deposit K48 polyubiquitin chains on its target proteins [28]. Here we observed that Akirin-V5 was also decorated by K48-polyubiquitin chains, but independently of Hyd (S5 Fig).

Collectively, these data suggest that upon immune challenge, Hyd physically interacts with Akirin through its catalytic HECT domain to decorate Akirin with K63-polyUb chains. We previously published that Akirin physically bridges the NF-κB factor Relish and Bap60, a core member of the SWI/SNF chromatin-remodeling complex [19]. To understand whether Akirin K63-polyubiquitination is instrumental for the interaction of Akirin with Relish or Bap60, we performed co-immunoprecipitation experiments in S2 cells depleted for Hyd and transfected with *Akirin-V5* and *Rel68-HA* or *BAP60-HA* (Fig 3D). As previously reported [19], Akirin-V5 co-precipitated either with the active form of the NF-κB homolog Relish (Rel68-HA) or with Bap60 (Bap60-HA) (Fig 3D). However, in the absence of Hyd, the interaction between Akirin-V5 and Rel68-HA was weakened (Fig 3D). Of note the interaction between Akirin-V5 and Bap60-HA is independent of Hyd (Fig 3D). Upon immune challenge, the transcriptional kinetic of Akirin-dependent and Akirin-independent genes was different. Akirin-independent genes (*AttD*, *Pgrp-LB*) were strongly transcribed after 1 hour, whereas Akirin-dependent genes (*AttA*, *AttC*) were strongly detectable after 3 hours (S6 Fig). Interestingly, the delayed activation of Akirin-dependent genes correlated with the robust K63-polyUb chains detection on Akirin at 3 hours after immune challenge (Fig 3B).

These results suggest that, upon immune challenge, Hyd is required to deposit K63-polyUb chains on Akirin for subsequent binding to the NF-κB factor Relish, and efficient transcription of Akirin-dependent genes.

### UBR5, the human ortholog of Hyd, is required for NF-κB transcriptional selectivity during the inflammatory response

**Fig 4:**
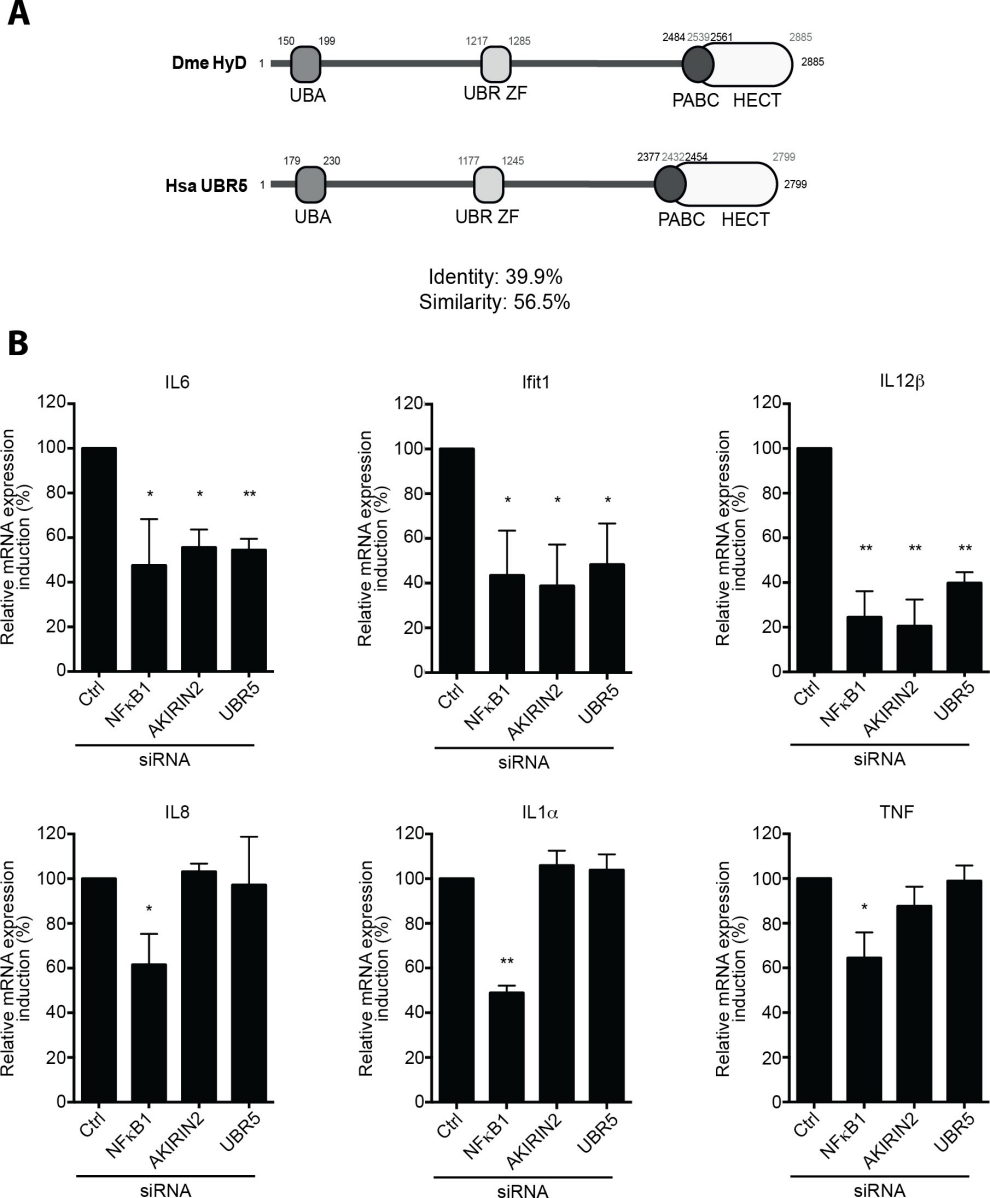
Hyd/UBR5 is necessary for NF-κB target genes activation. (**A**) Graphical representation of the predicted functional domains of *Drosophila melanogaster* Hyd and *Homo sapiens* UBR5. This representation highlights a conserved Ubiquitin-associated (UBA) domain, poly(A)-binding protein C-terminal (PABC) domain, Homologous to the E6-AP Carboxyl Terminus (HECT) domain and UBR type Zinc Finger (UBR ZF) domain. Annotation based InterPro software. Sequence similarity percentages calculated using the EMBOSS NEEDLE tool (default settings). (**B**) Quantitative RT-PCR of *IL6*, *Ifit1*, *IL12β*, *IL8*, *Il1α* and *TNF* mRNA from LPS-stimulated THP1 cells. They were transfected with scrambled siRNA (negative control) or siRNA targeting *NF-κB1*, *AKIRIN2* (positive controls) or *UBR5*. Data are represented as mean ± standard deviation of three independent experiments. After the ratio of stimulated over unstimulated values for each condition was determined, statistical significance was established by comparing genes knockdown with scrambled siRNA control. *P-value < 0.05; **P-value < 0.01; ***P-value < 0.001.

The Akirin-dependent molecular mechanism underlying the selective activation of NF-κB target genes is well conserved from *Drosophila* to mammals [18–20]. It is established that i) in human, AKIRIN2 is the functional homologue of *Drosophila* Akirin [18, 20], and ii) downstream of NF-κB pathways, AKIRIN2 operates a dichotomy between the AKIRIN2-dependent and -independent genes to be transcribed [20]. UBR5 is the human ortholog of the *Drosophila* E3-ubiquitin ligase Hyd [21, 28]. Comparison between UBR5 and Hyd revealed the presence of similar functional domains, as well as 39,9% of sequence identity and 56,5% of sequence similarity (Fig 4A and S7 Fig). Therefore, we addressed the potential requirement of UBR5 in NF-κB selective transcriptional response mediated by AKIRIN2 during the human inflammatory response. A previous study using human HeLa cells and mouse macrophages has identified AKIRIN2-dependent genes (such as *IL6*, *Ifit1*, and, *IL12β)*, and AKIRIN2-independent genes (such as *IL8*, *IL1α* and *TNF) [20].* Here, we depleted THP1 (a human monocytic cell line) or HeLa cell lines for either NF-κB1, AKIRIN2 or UBR5 by siRNA (using scrambled siRNA as controls). We first confirmed that, as in HeLA cells, the AKIRIN2-dependent genes (*IL6*, *Ifit1*, and *IL12β*) and AKIRIN2-independent genes (*IL8*, *IL1α* and *TNF*) conserved their transcriptional activation dichotomies in THP1 cells (Fig 4B, S8 Fig and S9 Fig). Lacking NF-κB1 in these human cell lines impaired NF-κB target genes activation upon LPS or IL-1β stimulation (Fig 4B, S8 Fig and S9 Fig). THP1 and HeLa cells depleted for AKIRIN2 or UBR5 showed a decreased level of AKIRIN2-dependent target gene (*IL6*, *Ifit1*, and, *IL12β*) activation when compared to control (Fig 4B, S8 Fig and S9 Fig). This result suggests a conserved function of UBR5 in the selective transcription of NF-κB target genes mediated by AKIRIN2. However, the precise mechanisms by which UBR5 impacts the transcription of AKIRIN2-dependent target genes remains to be explored.

**Fig 5:**
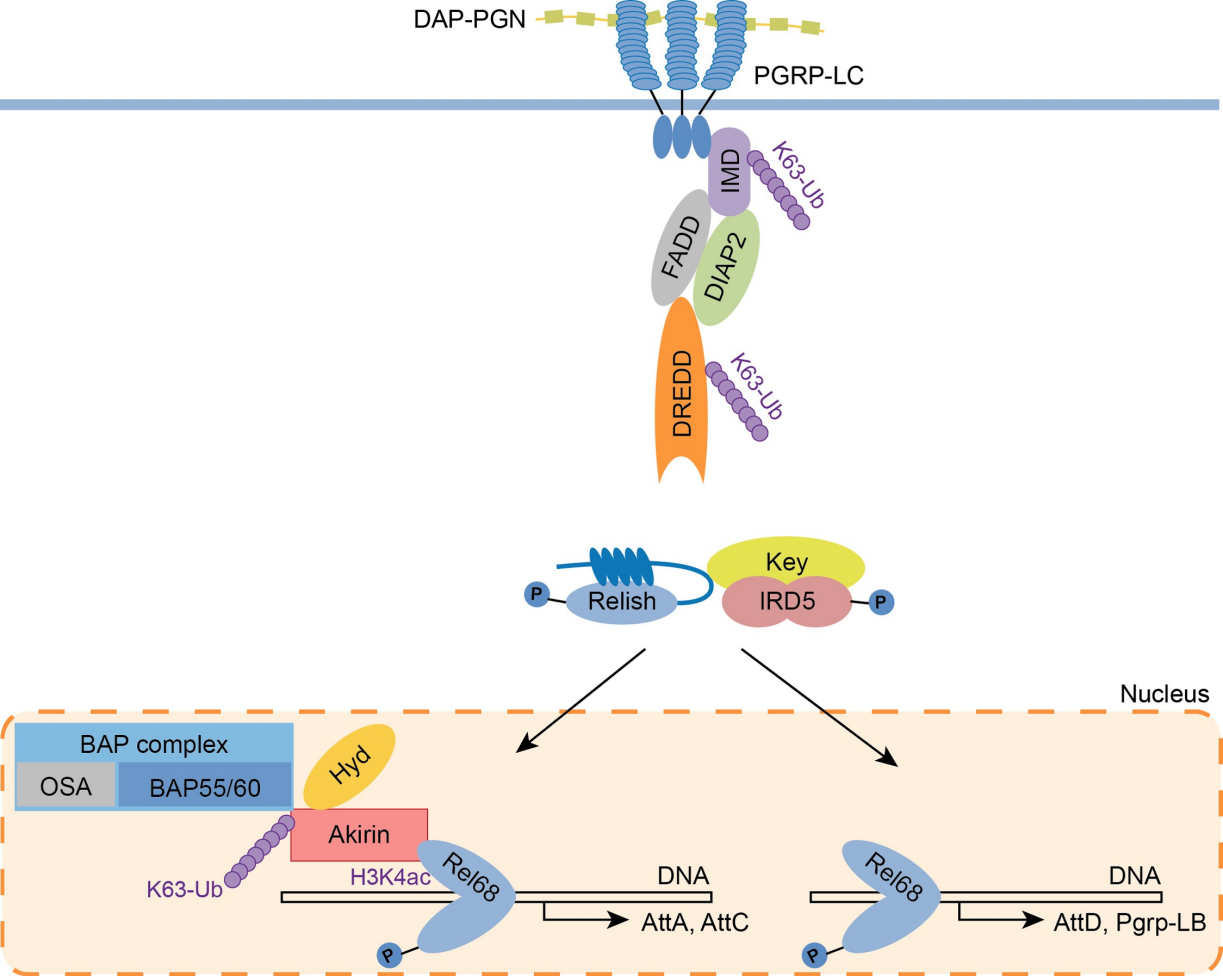
Schematic model of Hyd involvement in the IMD signaling pathway in *Drosophila*. Model showing the role of Hyd in the expression of the Akirin-dependent genes in the IMD pathway. After activation of the pathway, allowed by the K63-polyUb chains deposition on the complexes IMD and DREDD by the E3-ubiquitin ligase Diap2, Relish is translocated. Hyd is necessary for the K63-polyUb of the NF-κB co-factor Akirin and its interaction with the NF-κB factor Relish. This interaction is crucial for the expression of Akirin-dependent genes (like *Attacin-A* and *Attacin-C*), necessary for an adequate innate immune response. DAP-PGN: meso-diaminopimelic acid-type peptidoglycan of Gram-negative bacteria, P-tag: phosphorylation marks.

Taken altogether, our results show that the HECT-type E3 ubiquitin ligase Hyd/UBR5 is involved in NF-κB pathway regulation in *Drosophila* and mammals. In fruit fly, Hyd deposits K63-polyUb chains on Akirin and is required to bridge Akirin and the NF-κB factor Relish. This interaction is necessary for the transcription of an essential subset of NF-κB target genes, downstream of the IMD pathway (Fig 5).

## Discussion

Using *Drosophila* genetics, we describe here a function for the HECT E3 ubiquitin ligase Hyd in the innate immune response. Using human cell lines (HeLa and THP1), we could also show that this function of Hyd downstream of the NF-κB pathway is conserved in humans.

In both *Drosophila* and humans, NF-κB dependent signaling pathways are among the best-known examples of the role of ubiquitin linkage to target proteins in signal transduction [4, 29], ubiquitination being involved at every level of the NF-κB pathway, from membrane receptors to chromatin-associated proteins. In order to identify new E3-ubiquitin ligases involved in the *Drosophila* innate immune response, we conducted a RNAi-based screen. We showed, in addition to Diap2 already known to be a *bona fide* member of the IMD pathway [13, 14], that other RING-domain E3 ubiquitin ligases (CG5334, bon) were involved in the activation of the IMD pathway in *Drosophila* S2 cells after immune challenge. In addition, our results suggested that other RING-domain E3 ubiquitin ligases such as M-cup, Mkrin1 and Mura down-regulate IMD pathway target genes activation. This screen also suggested that a HECT E3-ubiquitin ligase, namely Hyd, is involved in the innate immune response.

In *Drosophila*, Hyd is reported to be located in the nuclear and in the cytoplasmic fraction of cells to participate in various phenomena during development such as cellular proliferation [30, 31]. More precisely, Hyd shapes hedgehog signaling by differentially restraining the transcriptional activity of Cubitus interuptus via selective association with respective promoters [27]. More recently, Hyd and its orthologue UBR5 were reported to act at the level of Wnt signaling target gene promoter to enable gene transcription [28]. Here we identified the HECT E3 ubiquitin-ligase Hyd in *Drosophila* as responsible for the ubiquitination of Akirin and its subsequent binding to the NF-κB transcription factor Relish. In addition, identification of protein interactors for AKIR-1, the *Caenorhabditis elegans* homologue of *Drosophila* Akirin, also revealed Hyd/UBR5 [32]. Altogether these results point to a conserved function of the HECT E3 ubiquitin ligase Hyd/UBR5 as nuclear selector for gene activation.

Downstream of the IMD pathway, the NF-κB transcription factor Relish target genes could be divided into two subgroups, Akirin-dependent and Akirin-independent genes [19]. Targeting *Hyd* by RNAi in *Drosophila* S2 cells dampened the activation of Akirin-dependent genes upon immune stimulation. Depleting Hyd from *Drosophila* fat-body prevents fly survival to immune challenge with the Gram-negative bacteria *E*. *coli*, demonstrating the biological relevancy of its function. Co-immunoprecipitation experiments showed that Hyd interacts with Akirin through its catalytic domain to deposit K63-polyUb chains. Of note, the K63-polyubiquitination of Akirin by Hyd is performed only after immune challenge, suggesting that an immune-triggered signal governs this event and remains to be explored. Because our observations are based on overexpressed proteins, it would be of interest to evaluate if endogenous Akirins are K63-polyubiquitinated by Hyd upon immune challenge. Additionally, it is still unclear how the K63-polyubiquitin chains on Akirin physically interact with Relish to set a bridge, as no Ubiquitin Binding Domain (UBD) have been described for Relish. During development, Akirin is also known to be able to link another transcription factor, Twist [33]. It remains to be investigated if Hyd affects Akirin-Twist dependent transcriptional response.

The HECT Ubiquitin ligase family is known in mammals and *Drosophila* to regulate biological phenomena [11]. We found that the mammalian ortholog of Hyd, UBR5 [21] is involved in NF-κB transcriptional selective response in human cell lines as well. This suggests a conserved role for Hyd/UBR5 on AKIRIN2, even though we do not know if AKIRIN2 is ultimately ubiquitinated. A dedicated study of UBR5 role in NF-κB pathways is needed to completely assess it. It is known that UBR5 inhibits the TNF receptor associated factor 3 (Traf3) [34], an inhibitor of the NF-κB pathway [35]. Thus, the role of UBR5 might be indirect.

When *Hyd* or *UBR5* was attenuated, only a subset of NF-κB target genes is expressed, diminishing the intensity of the innate immune response in *Drosophila* and inflammatory response in mammals, similarly to the inactivation of Akirin [19, 20]. The link between excessive activation of NF-κB signaling pathways during e.g chronic inflammation and cancer progression or appearance is now on the spotlight [36]. Uncontrolled activation of NF-κB due to deregulation of ubiquitin-ligases has been reported in many diseases [37] and UBR5 has been described to be involved in several types of cancer in humans [38]. Our findings point to the HECT E3-ubiquitin ligase UBR5 as an interesting drug target to modulate NF-κB signaling, control the development of inflammatory diseases and potentially improve treatments for cancer.

## Material and methods

### Cell culture

S2 cells were cultured at 25°C in Schneider's medium (Biowest) supplemented with 10% fetal (vol/vol) calf serum (FCS), penicillin/streptomycin (50 μg/ml of each) and 2 mM glutamax. HeLa cell line (gift from IGBMC, Illkirch, France) was cultured in DMEM containing 10% (vol/vol) FCS, 40 μg/mL gentamycin. THP1 cell line (ATCC-TIB-202) was cultured in RPMI containing 10% (vol/vol) FCS and penicillin/streptomycin (50 μg/ml of each).

### dsRNA E3 ubiquitin ligases screen

A list containing 174 E3 ubiquitin ligases in the *Drosophila* genome, consisting predominantly of HECT, RING, and U-box proteins was curated manually by GO- and protein domain-term search in Flybase FB2012_06 Dmel Release 5.48 [22]. Based on this list, a *Drosophila* E3 ligase dsRNA library was generated in Michael Boutros’s laboratory [39], listed in S2 Table. The screen experiments were performed using 1F3 cells stably expressing AttA firefly luciferase [17]. Two days after transfection with an Actin renilla luciferase construct, cells were collected and distributed into 96-well screening plates at a density of 4.5 x 10^4^ cells per well. Cells were transfected with 3 μg of each dsRNA in the *Drosophila* E3-ubiquitin ligase dsRNA library in triplicate by bathing method as previously described [19]. At day 5 post-transfection, cells were stimulated for 48 h with heat-killed *E*. *coli* (40:1) before being lysed. Luciferase activity was quantified in a luminometer (Mithras LB940, Berthold) after addition of the substrate (Dual luciferase assay kit, Promega).

### RNA interference

The double-strand RNAs for the knockdown experiments in *Drosophila* cells were prepared according to [19]. Fragments for the different genes were generated from genomic DNA templates using oligonucleotides designed for use with Genome-RNAi libraries [40] and are listed in S3 Table. The small interfering RNAs used for the knockdown experiment in mammalian cell lines cells were purchased from Ambion (S4 Table).

### Plasmid constructs

*pAC-Akirin*, *pAC-Akirin-V5*, *pAC-Pgrp-LC*, *pAC-Imd*, *pMT-Rel-HA*, *pMT-Bap60-HA* and *pAC-Gal4* constructs were described previously [18, 19]. *pUAS-Hyd-V5* and *pUAS-Hyd-CS-V5* were kindly provided by Xinhua Lin laboratory [27].

### Cell transfection

*Drosophila* S2 cells were transfected with double-strand RNAs using the bathing method described in [19] or with plasmids using the Effectene transfection kit (Qiagen). HeLa cells were transfected with siRNA using Lipofectamine RNAiMax Transfection Reagent (Invitrogen). THP1 cells were transfected with siRNA using Lipofectamine 3000 Transfection Reagent (Invitrogen).

### RNA extraction and quantification

For the *in vitro* experiments, RNA was extracted from cells and treated with DNAse, using RNA Spin kit (Macherey Nagel). For the *in vivo* experiments, the procedure was done accordingly to [19]. Similarly, reverse-transcription and quantitative RT-PCR were performed as indicated in [19]. The levels of expression of genes of interest were normalized against the measured level of the RNA coding determined in each sample for ribosomal protein-49 in the case of *Drosophila* experiments and for GAPDH for HeLa and THP1 cells. Primers used for quantitative RT-PCR are listed in S5 Table.

### Isolation of nuclear and cytoplasmic cell fractions

Nuclear/cytoplasmic fractionation was performed using the NE-PER Nuclear and Cytoplasmic Extraction Reagents kit (Thermo Scientific), following the manufacturer’s instructions. The cell pellet was suspended in 500 μl of cytoplasmic extraction reagent I. After a 10 min incubation on ice, 11 μl of cytoplasmic extraction reagent II was added. The supernatant fraction was then collected after a 5 min centrifugation at 16 000 g. The nuclear fraction was collected following the suspension of the nuclear pellet in 250 μl of Nuclear extraction reagent, incubation for 40min on ice and centrifugation for 10 min at 16 000g.

### Immunoprecipitation and Western blot

Cells were treated for the indicated times with heat-killed *E*. *coli* (40:1) at 25°C. The cells were harvested, washed in PBS and lysed in 200 μl of Pierce IP Lysis Buffer (Thermo Scientific), with protease inhibitor cocktail (Roche). Immunoprecipitations were performed overnight at 4°C based on [19, 41], with either rabbit polyclonal anti-Akirin [19] or anti-ubiquitin Lys63 specific antibodies (Millipore 05–1308), or anti-V5 (Merck V8137) coupled with Dynabeads Protein G (Invitrogen) and anti-V5 or anti-HA antibodies coupled to agarose beads (Sigma). Proteins were detected by Western blotting using anti-Akirin, anti-ubiquitin Lys63 specific, anti-ubiquitin Lys48 specific (Merck 05–1307), anti-Ubiquitin (SantaCruz Biotechnology P4D1), anti-H3 histone (Abcam ab1791), anti-RpS15 (Abcam ab157193), anti-V5 (Invitrogen r96025) and anti-HA (Abcam ab9110) antibodies.

### Fly strains

Stocks were raised on standard cornmeal-yeast-agar medium at 25°C with 60% humidity. To generate conditional knockdown in adult flies, we used the GAL4-GAL80^ts^ system [26]. Fly lines carrying a *UAS-RNAi* transgene targeting *relish* (108469), *akirin* (109671), and *hyd* (44675, named here Hyd1; 44676, named here Hyd2) were obtained from the Vienna Drosophila RNAi Center (http://stockcenter.vdrc.at/control/main). Fly line carrying a *UAS-RNAi* transgene against *GFP* (397–05) was obtained from the Drosophila Genetic Resource Center (Kyoto, Japan; http://www.dgrc.kit.ac.jp/index.html). *UAS-RNAi* flies were crossed with *Actin-GAL4/CyO*; *Tub-GAL80ts* flies at 18°C. Emerged adult flies were then transferred to 29°C to activate the *UAS-GAL4* system for 6–7 days. Nine-day-old flies were used—three batches of twenty females for survival assays and ten males for quantitative RT-PCR experiments.

### Immune challenge

*Drosophila* S2 cells were stimulated with heat-killed *E*. *coli* (40:1) [42]. HeLa cells were stimulated with recombinant human IL-1β (10 ng/ml) for 4h. THP1 cells were stimulated with Lipopolysaccharide (LPS) Solution (1 μg/ml) for 4h. IL-1β and LPS were purchased from Invitrogen. Microbial challenges were performed by pricking adult flies with a sharpened tungsten needle dipped into PBS or concentrated *E*. *coli* strain DH5aGFP bacteria solution at 25°C, for either several days (for survival assays) or 6h (for quantitative RT-PCR experiments) [19, 42]. Bacteria were grown in Luria broth (LB) at 37°C.

### Bioinformatic analysis of HyD and UBR5

Amino acid sequences of *Drosophila melanogaster* HyD (ID: P51592) and *Homo sapiens* UBR5 (ID: O95071) were retrieved from UniProt (uniport.org). Predicted functional domains were annotated using the InterPro database (v77.0) from the European Molecular Biology Laboratory [43]. In order to calculate identity and similarity percentages, sequences were aligned to each other using the EMBOSS NEEDLE tool [44], also from the European Molecular Biology Laboratory, under default settings. Graphical visualization of the similarity percentage between HyD and UBR5 alongside the amino acid sequence was performed with the LALNVIEW software [45] after sequence alignment with the SIM–Local similarity program [46] under default settings.

### Statistical analysis

P values for quantitative RT-PCR were calculated using the two-tailed unpaired Student t test using GraphPad Prism version 6.0c for Mac, GraphPad Software, San Diego, California USA. Log-rank analyses of survival assay were performed using RStudio version 1.1.463 and the function survdiff of the survival package (version 2.43–3; Terry M Therneau, 2018). RStudio Team (2016). RStudio: Integrated Development for R. RStudio, Inc., Boston, MA USA.

## Supporting information

S1 Fig Knockdown efficiency of the double strand RNA used in *Drosophila* S2 cells. (DOCX)

S2 Fig Knockdown efficiency of the Gal4-UAS system used in adult flies. (DOCX)

S3 Fig Interaction assay between Diap2 and Akirin. (DOCX)

S4 Fig Ubiquitinated Akirin accumulates in nuclear cell fraction after immune challenge. (DOCX)

S5 Fig Akirin is K48-polyubiquitinated independently of Hyd. (DOCX)

S6 Fig Kinetic of immune induced genes at different hours after stimulation. (DOCX)

S7 Fig *Drosophila* HyD and Human UBR5 share a high sequence similarity. (DOCX)

S8 Fig Knockdown efficiency in HeLa cells of the small interfering RNA used in mammalian cell lines. (DOCX)

S9 Fig Hyd/Ubr5 is necessary for activation of a subset of NF-κB target genes in HeLa cells. (DOCX)

S1 Table Induction of Attacin-A after knockdown of the luciferase screen candidates in *Drosophila* S2 cells. (DOCX)

S2 Table Double strand RNA sequences used in the luciferase screen in *Drosophila* S2 cells. (XLSX)

S3 Table Oligonucleotides used to generate double strand RNA in *Drosophila* S2 cells. (DOCX)

S4 Table References of small interfering RNA used in mammalian HeLa cells. (DOCX)

S5 Table Oligonucleotides used for quantitative real-time PCR. (DOCX)
