# Correction: Hyd ubiquitinates the NF-κB co-factor Akirin to operate an effective immune response in *Drosophila*

**DOI:** 10.1371/journal.ppat.1013785

**Published:** 2025-12-18

**Authors:** Alexandre Cammarata-Mouchtouris, Xuan-Hung Nguyen, Adrian Acker, François Bonnay, Akira Goto, Amir Orian, Marie-Odile Fauvarque, Michael Boutros, Jean-Marc Reichhart, Nicolas Matt

Following the publication of this article [1], the corresponding author contacted PLOS about Figs 3A and S6.

Specifically, the corresponding author stated that they have been unable to reproduce the results shown in Fig S6 in repeat experiments carried out after the publication of [1]. They stated that the cause of this irreproducibility remains unclear, and they cannot confirm the conclusions drawn from Fig S6. Therefore, the sentence: ‘Akirin-independent genes (*AttD, Pgrp-LB*) were strongly transcribed after 1 hour, whereas Akirin-dependent genes (*AttA, AttC*) were strongly detectable after 3 hours (S6 Fig). Interestingly, the delayed activation of Akirin-dependent genes correlated with the robust K63-polyUb chain detection on Akirin at 3 hours after immune challenge (Fig 3B)’ in the ‘Hyd-mediated K63-polyubiquitination of Akirin is critical for Akirin binding to Relish’ section of the Results is no longer supported.

PLOS considers that the overall conclusions of this article [1] remain unaffected.

The corresponding author stated that, while verifying the remaining data in [1] in response to the above concerns with Fig S6, they were unable to carry out repeat experiments for Fig 3A due to the loss of plasmids necessary for the experiment. They also stated that Figs 3B-D were generated independently of Fig 3A. PLOS have not identified any concerns with Fig 3 in [1].

The underlying data were not included with the original article [1]. The corresponding author provided the original quantitative data files in support of [1] in S1-S2 Files. With this Correction, all relevant data are now provided.

## Supporting information

S1 File The original underlying quantitative data and images in support of Figs 1–4. (ZIP)

S2 File The original underlying quantitative data in support of Figs S1-2 and S8-9, and the original images for Fig S3. (ZIP)
