# Supplementary material for: Hyd ubiquitinates the NF-κB co-factor Akirin to operate an effective immune response in Drosophila
Source: PLoS Pathog. 2020 Apr 27;16(4):e1008458. doi: 10.1371/journal.ppat.1008458 (PMC7205318; doi:10.1371/journal.ppat.1008458)
Supplement: S1 Fig — (DOCX) [file ppat.1008458.s001.docx]

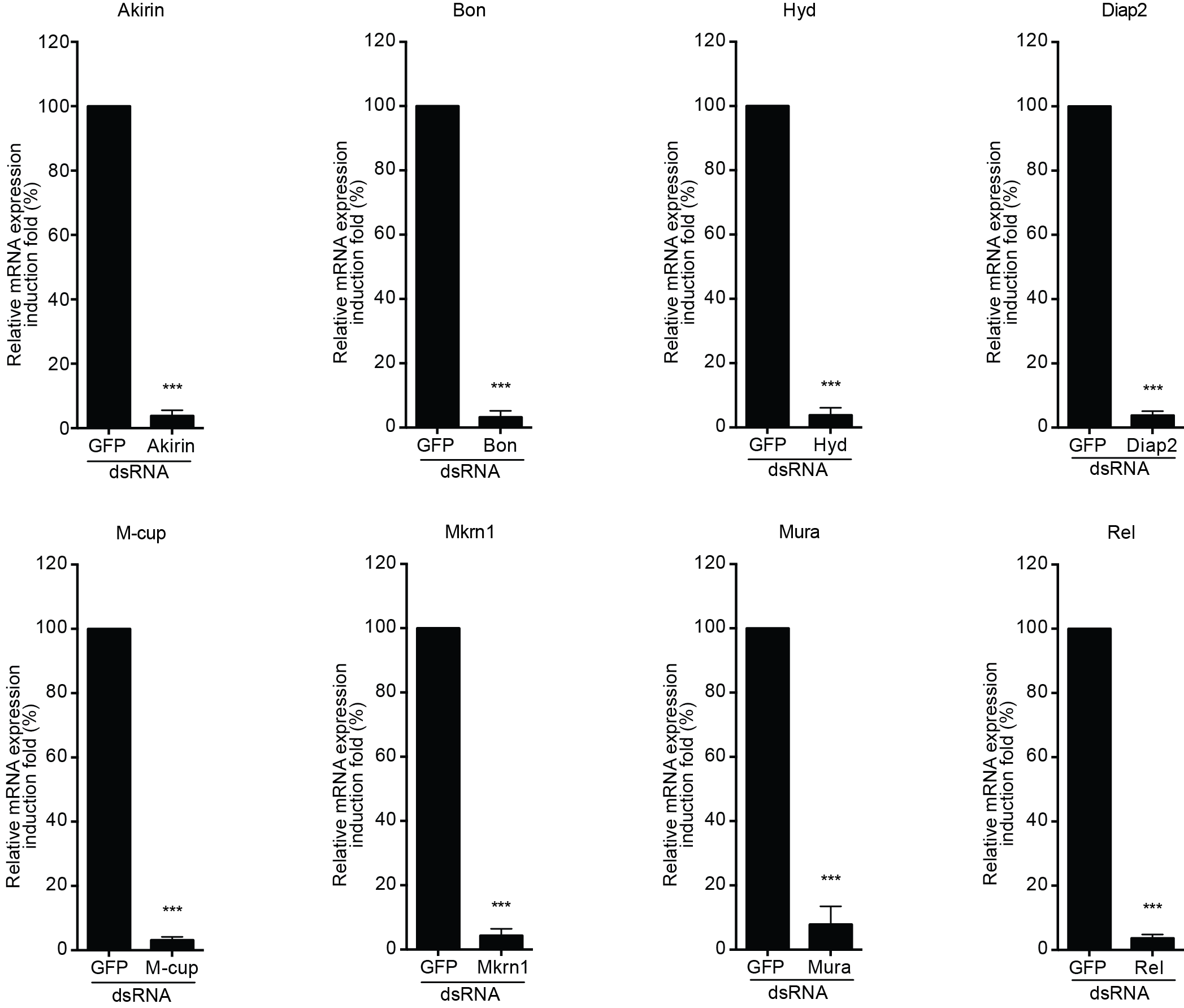


**S1 Fig. Knockdown efficiency of the double strand RNA used in *Drosophila* S2 cells.** Quantitative RT-PCR of *Akirin*, *Bon*, *Hyd*, *Diap2*, *M-cup*, *Mkrn1*, *Mura* and *Relish* mRNA from S2 cells transfected with dsRNA targeting *GFP* and the respective genes.

Data are represented as mean ± standard deviation of three independent experiments. Statistical significance was established by comparing genes knockdown with *GFP* dsRNA. *P-value < 0.05; **P-value < 0.01; ***P-value < 0.001.
