# Supplementary material for: Hyd ubiquitinates the NF-κB co-factor Akirin to operate an effective immune response in Drosophila
Source: PLoS Pathog. 2020 Apr 27;16(4):e1008458. doi: 10.1371/journal.ppat.1008458 (PMC7205318; doi:10.1371/journal.ppat.1008458)
Supplement: S2 Fig — (DOCX) [file ppat.1008458.s002.docx]

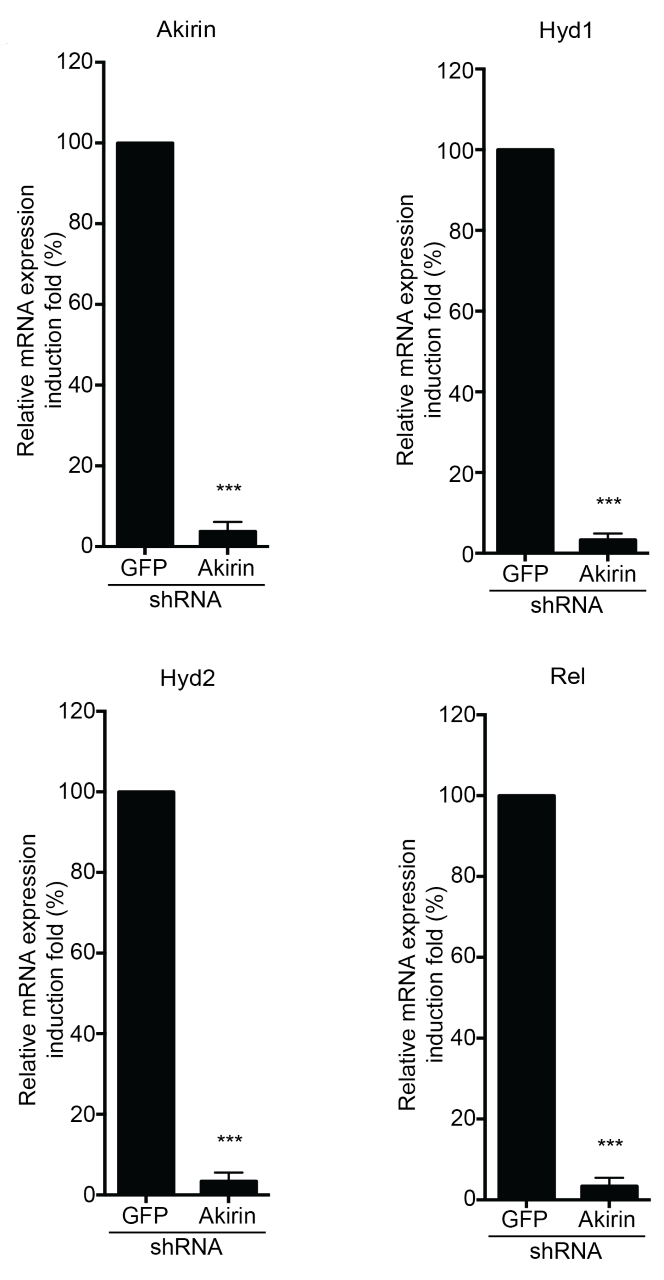


**S2 Fig. Knockdown efficiency of the Gal4-UAS system used in adult flies.**

Quantitative RT-PCR of *Akirin*, *Hyd* and *Relish* mRNA from the adult fly lines in which the Gal4-UAS system was used to knockdown the respective genes (two lines for *Hyd*).

Data are represented as mean ± standard deviation of three independent experiments. Statistical significance was established by comparing genes knockdown with *GFP* siRNA. *P-value < 0.05; **P-value < 0.01; ***P-value < 0.001.
