# Supplementary material for: Hyd ubiquitinates the NF-κB co-factor Akirin to operate an effective immune response in Drosophila
Source: PLoS Pathog. 2020 Apr 27;16(4):e1008458. doi: 10.1371/journal.ppat.1008458 (PMC7205318; doi:10.1371/journal.ppat.1008458)
Supplement: S3 Fig — (DOCX) [file ppat.1008458.s003.docx]

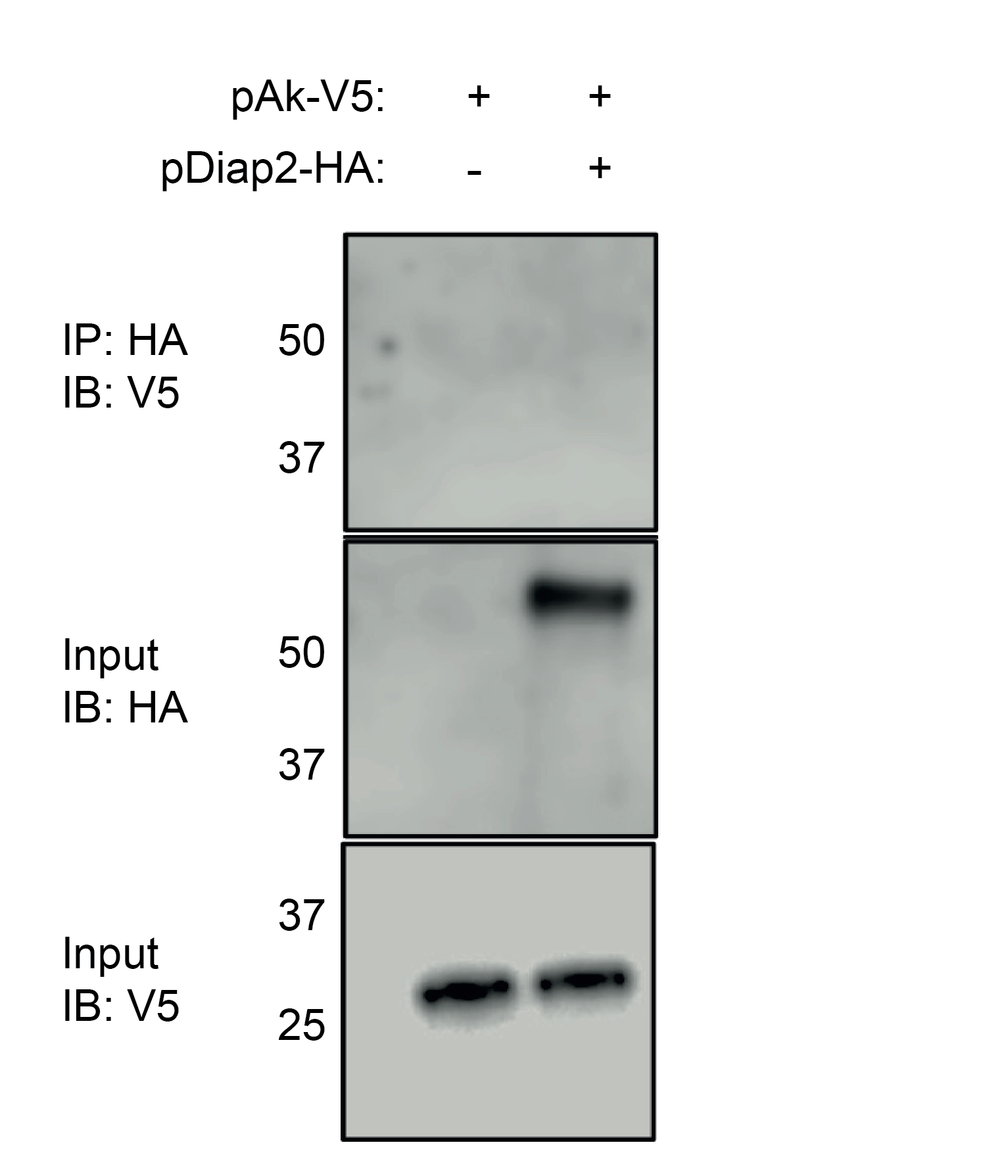


**S3 Fig. Interaction assay between Diap2 and Akirin.**

Co-immunoprecipitation assay between over-expressed *Diap2* and *Akirin* in S2 cells. The cells were transiently transfected with *Akirin-V5* and/or *Diap2-HA* expressing plasmids. Cell lysates were immunoprecipitated with anti-V5 coupled agarose beads. Immunoprecipitates were analyzed by Western blotting with anti-HA or anti-V5 antibodies.

Data are representative of 2 independent experiments.
