# Supplementary material for: Hyd ubiquitinates the NF-κB co-factor Akirin to operate an effective immune response in Drosophila
Source: PLoS Pathog. 2020 Apr 27;16(4):e1008458. doi: 10.1371/journal.ppat.1008458 (PMC7205318; doi:10.1371/journal.ppat.1008458)
Supplement: S4 Fig — (DOCX) [file ppat.1008458.s004.docx]

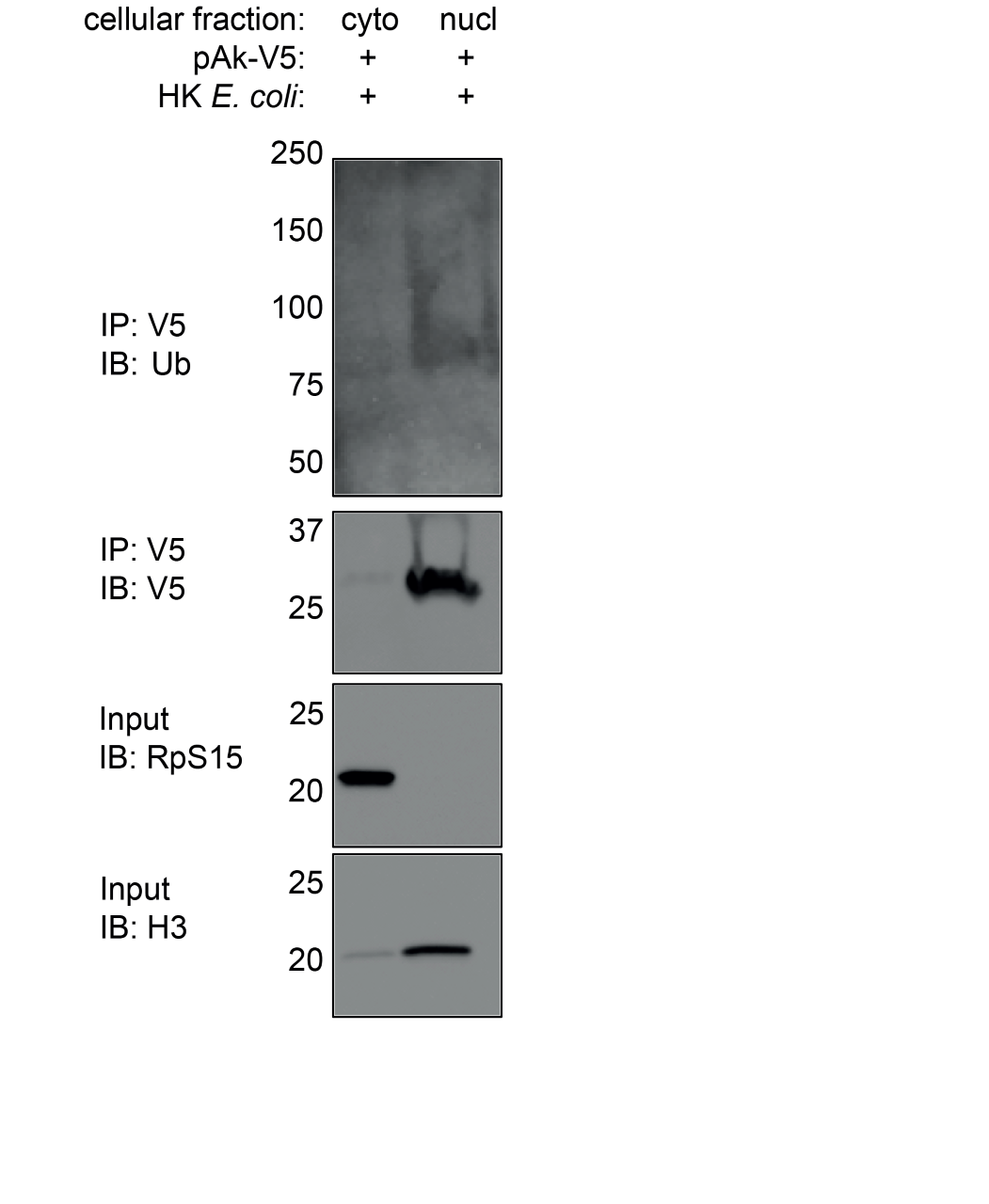


**S4 Fig. Ubiquitinated Akirin accumulates in nuclear cell fraction after immune challenge.**

Immunoprecipitation assay of Akirin after immune challenge. S2 cells were transiently transfected with *Akirin-V5* expressing plasmid. Nuclear and cytoplasmic cell fractions were isolated and lysates were immunoprecipitated with anti-V5 coupled agarose beads. Immunoprecipitates were analyzed by Western blotting with anti-polyUb, anti-V5, anti-RpS15 and anti-H3 antibodies.

Data are representative of 2 independent experiments.
