# Supplementary material for: Hyd ubiquitinates the NF-κB co-factor Akirin to operate an effective immune response in Drosophila
Source: PLoS Pathog. 2020 Apr 27;16(4):e1008458. doi: 10.1371/journal.ppat.1008458 (PMC7205318; doi:10.1371/journal.ppat.1008458)
Supplement: S5 Fig — (DOCX) [file ppat.1008458.s005.docx]

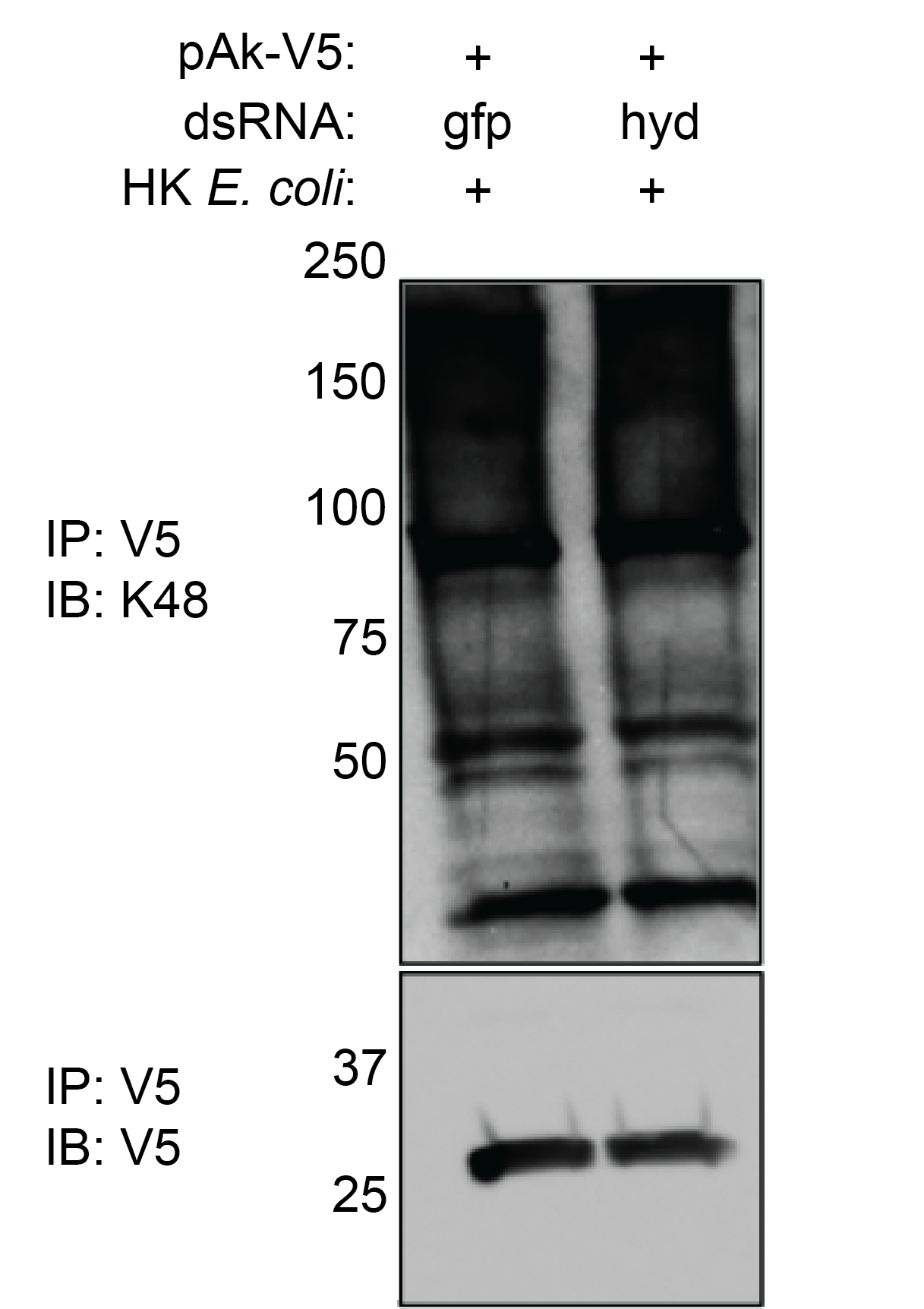


**S5 Fig. Akirin is K48-polyubiquitinated independently of Hyd.**

Immunoprecipitation assay of Akirin after immune challenge. S2 cells were transiently transfected with *Akirin-V5* expressing plasmid and dsRNA targeting *GFP* or *Hyd*. Cell lysates were immunoprecipitated with anti-V5 coupled agarose beads. Immunoprecipitates were analyzed by Western blotting with anti-K48-polyUb and anti-V5 antibodies.

Data are representative of 2 independent experiments.
