# Supplementary material for: Hyd ubiquitinates the NF-κB co-factor Akirin to operate an effective immune response in Drosophila
Source: PLoS Pathog. 2020 Apr 27;16(4):e1008458. doi: 10.1371/journal.ppat.1008458 (PMC7205318; doi:10.1371/journal.ppat.1008458)
Supplement: S6 Fig — (DOCX) [file ppat.1008458.s006.docx]

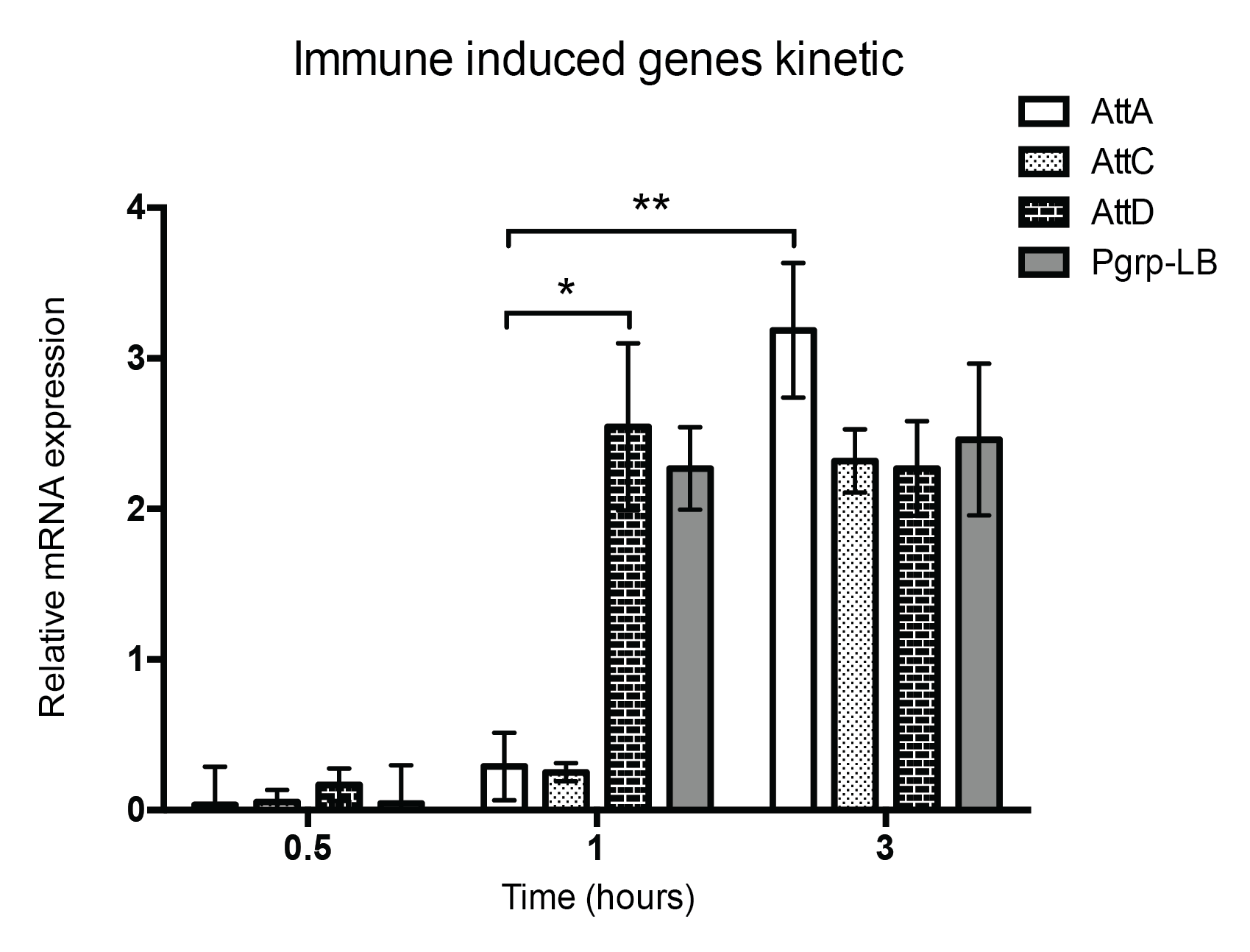


**S6 Fig. Kinetic of immune induced genes at different hours after stimulation.**

Quantitative RT-PCR of *Attacin-A*, *Attacin-C*, *Attacin-D* and *Pgrp-LB* mRNA from HKE-stimulated S2 cells.

Data are represented as mean ± standard deviation of three independent experiments. Statistical significance was established using *Attacin-A* expression at 1h as a control. *P-value < 0.05; **P-value < 0.01; ***P-value < 0.001.
