# Supplementary material for: Hyd ubiquitinates the NF-κB co-factor Akirin to operate an effective immune response in Drosophila
Source: PLoS Pathog. 2020 Apr 27;16(4):e1008458. doi: 10.1371/journal.ppat.1008458 (PMC7205318; doi:10.1371/journal.ppat.1008458)
Supplement: S7 Fig — (DOCX) [file ppat.1008458.s007.docx]

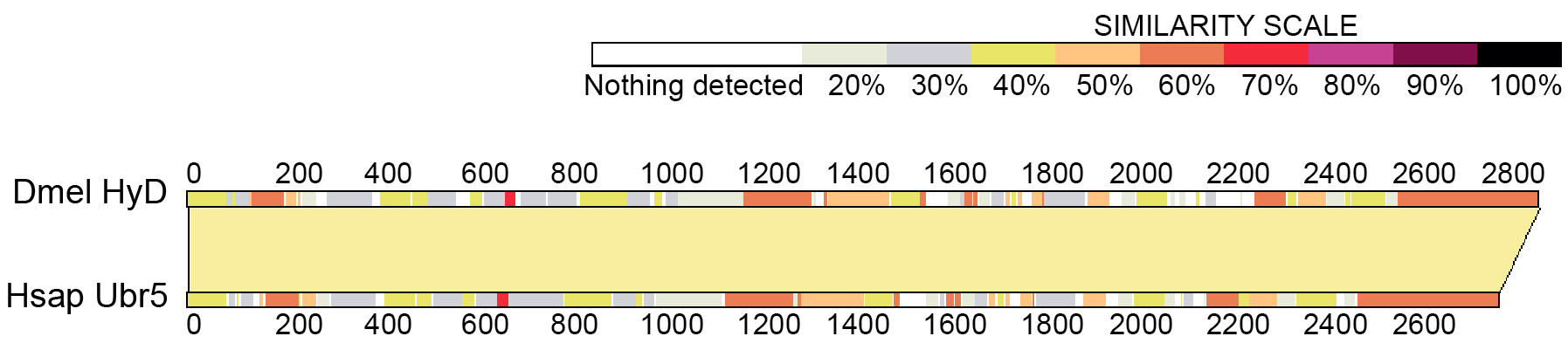


**S7 Fig. *Drosophila* Hyd and Human UBR5 share a high sequence similarity.**

Sequence comparison between *Drosophila melanogaster* Hyd (Dmel HyD) and *Homo sapiens* UBR5 (Hsap Ubr5). High conservation between Hyd and UBR5 can be observed, in particular at the position of the putative protein domains. Of note, the position of the predicted functional domains are: i) UBA: *Dmel* 150-199 *Hsap* 179-230; ii) UBR ZF: *Dmel* 1217-1285 *Hsap* 1177-1245; iii) PABC *Dmel* 2484-2561 *Hsap* 2377-2464; iv) HECT *Dmel* 2539-2885 *Hsap* 2432-2799. Graphical visualization performed with the LALNVIEW program.
