# Supplementary material for: Hyd ubiquitinates the NF-κB co-factor Akirin to operate an effective immune response in Drosophila
Source: PLoS Pathog. 2020 Apr 27;16(4):e1008458. doi: 10.1371/journal.ppat.1008458 (PMC7205318; doi:10.1371/journal.ppat.1008458)
Supplement: S8 Fig — (DOCX) [file ppat.1008458.s008.docx]

**
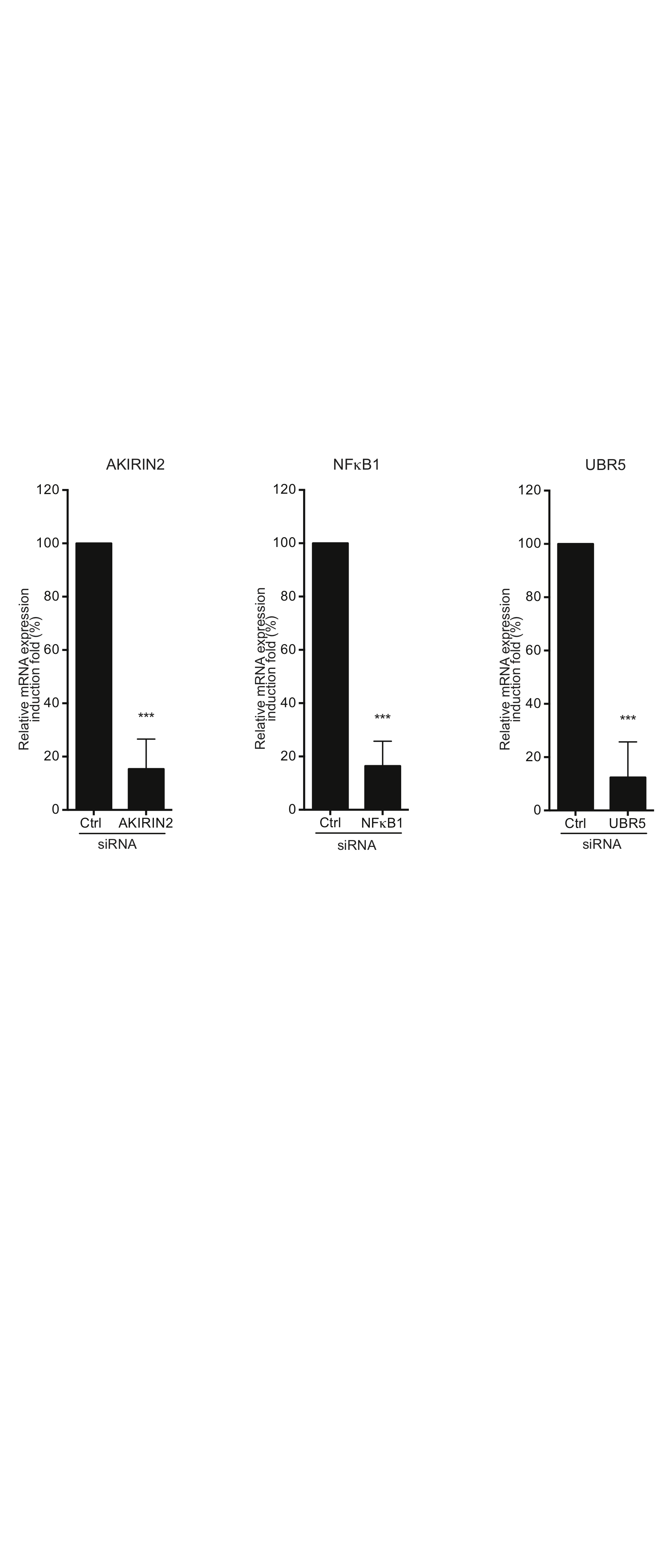
**

**S8 Fig. Knockdown efficiency in HeLa cells of the small interfering RNA used in mammalian cell lines.**

Quantitative RT-PCR of *AKIRIN2*, *NFκB1* and *UBR5* mRNA from HeLa cells transfected with siRNA scrambled and targeting the respective genes.

Data are represented as mean ± standard deviation of three independent experiments. Statistical significance was established by comparing genes knockdown with scrambled siRNA control. *P-value < 0.05; **P-value < 0.01; ***P-value < 0.001.
