# Supplementary material for: Hyd ubiquitinates the NF-κB co-factor Akirin to operate an effective immune response in Drosophila
Source: PLoS Pathog. 2020 Apr 27;16(4):e1008458. doi: 10.1371/journal.ppat.1008458 (PMC7205318; doi:10.1371/journal.ppat.1008458)
Supplement: S9 Fig — (DOCX) [file ppat.1008458.s009.docx]

**
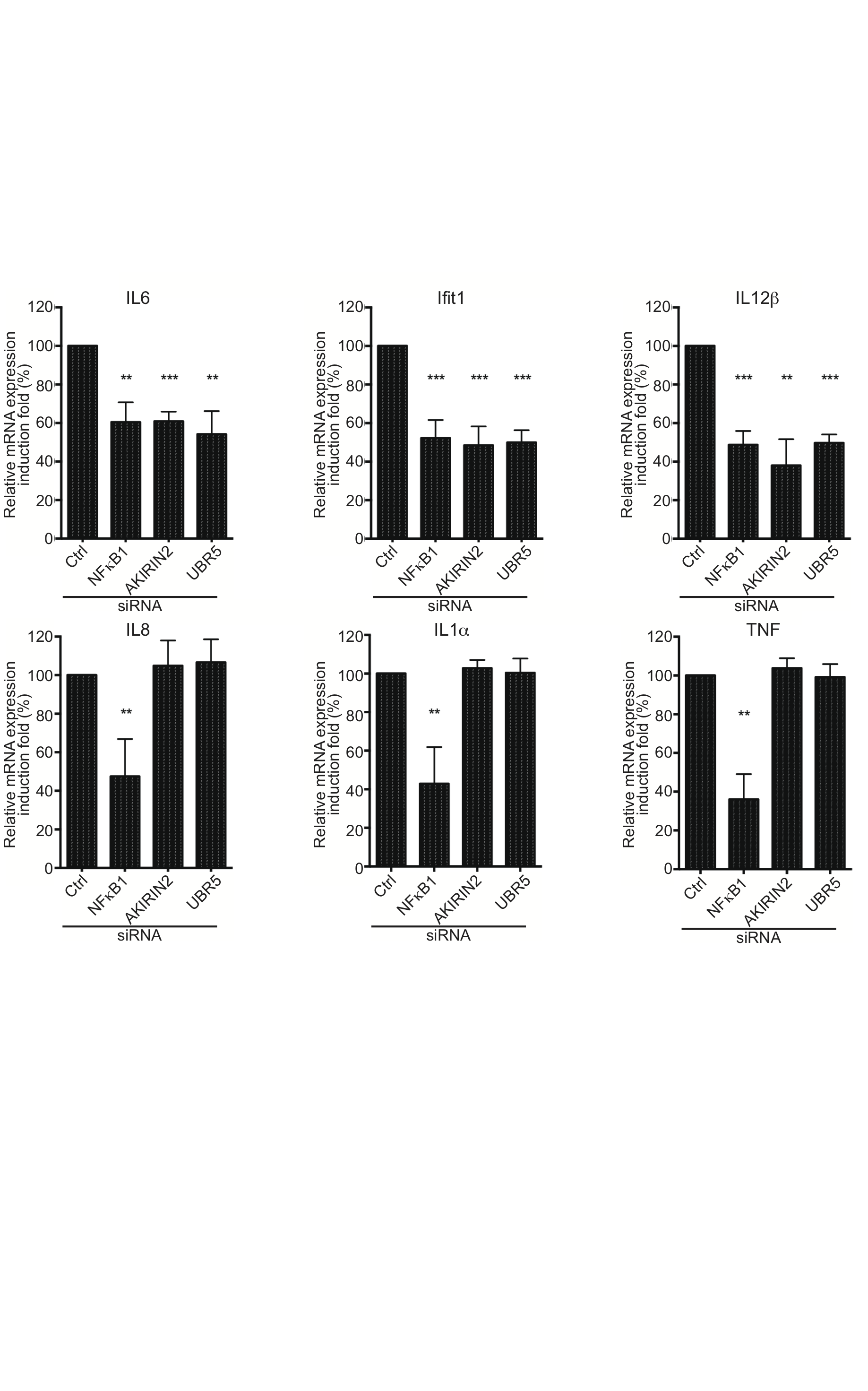
**

**S9 Fig. Hyd/UBR5 is necessary for activation of a subset of NF-κB target genes in HeLa cells.**

Quantitative RT-PCR of *IL6*, *Ifit1*, *IL12β*, *IL8*, *Il1α* and *TNF* mRNA from IL-1β-stimulated HeLa cells. They were transfected with scrambled siRNA (negative control) or siRNA targeting *NFκB1,* *AKIRIN2* (positive controls) or *UBR5*.

Data are represented as mean ± standard deviation of three independent experiments. After the ratio of stimulated with unstimulated values for each condition was determined, statistical significance was established by comparing genes knockdown with scrambled siRNA control. *P-value < 0.05; **P-value < 0.01; ***P-value < 0.001.
