# Supplementary material for: Hyd ubiquitinates the NF-κB co-factor Akirin to operate an effective immune response in Drosophila
Source: PLoS Pathog. 2020 Apr 27;16(4):e1008458. doi: 10.1371/journal.ppat.1008458 (PMC7205318; doi:10.1371/journal.ppat.1008458)
Supplement: S1 Table — (DOCX) [file ppat.1008458.s010.docx]

**Table S1. Induction of *Attacin-A* after knockdown of the luciferase screen candidates in *Drosophila* S2 cells.**

174 Drosophila E3 ubiquitin ligases were knocked down in S2 cells, after being transfected with the *Attacin-A–luciferase* reporter gene. This list presents the induction of *Attacin-A* in those cells after heat killed *E. coli* stimulation.
