# Supplementary material for: Hyd ubiquitinates the NF-κB co-factor Akirin to operate an effective immune response in Drosophila
Source: PLoS Pathog. 2020 Apr 27;16(4):e1008458. doi: 10.1371/journal.ppat.1008458 (PMC7205318; doi:10.1371/journal.ppat.1008458)
Supplement: S3 Table — (DOCX) [file ppat.1008458.s012.docx]

**Table S3. Oligonucleotides used to generate double strand RNA in *Drosophila* S2 cells.**

Are indicated: gene reference, dsRNA reference (http://www.genomernai.org/GenomeRNAi/), forward and reverse primers (without T7 promoter sequence TTAATACGACTCACTATAGG) used to produce T7 DNA matrix PCR product and PCR product size.

| **Gene** | **dsRNA reference** | **Forward** | **Reverse** |
| --- | --- | --- | --- |
| *Relish* | DRSC37194 | TGCCATGTGGAGTGCATTAT | TGCCATGTGGAGTGCATTAT |
| *Akirin* | DRSC26196 | ATCTTCCATCTGCAGCATCC | ACGGACTAGGTTCGGTGCTA |
| *Hyd* | DRSC28294 | GCGACCGAATAAGTCCAGAG | GCCACACGACCAGAGGTTAT |
| *Bon* | DRSC38123 | AGCCAGAAGTCGAAGGTGAA | TTGCTCAGACTCAGCGAAGA |
| *Diap2* | DRSC38402 | AAATCCATGTGATCTGCGGT | CCAGTGTAGCCAATTGTCCC |
| *M-cup* | DRSC28310 | GCGACCGAATAAGTCCAGAG | GCCACACGACCAGAGGTTAT |
| *Mkrn1* | BKN24610 | GATTGGTGTGTGCGTTTCAC | ATCGGCGAGATTATCATTGG |
| *Mura* | DRSC26645 | ATCTGGGTTTTGAGTGACCG | ATGAGTGATCGGGACAGAGG |
