# Supplementary material for: Hyd ubiquitinates the NF-κB co-factor Akirin to operate an effective immune response in Drosophila
Source: PLoS Pathog. 2020 Apr 27;16(4):e1008458. doi: 10.1371/journal.ppat.1008458 (PMC7205318; doi:10.1371/journal.ppat.1008458)
Supplement: S4 Table — (DOCX) [file ppat.1008458.s013.docx]

**Table S4. References of small interfering RNA used in mammalian HeLa cells.**

| **Gene** | **UniGene ID** | **siRNA ID** |
| --- | --- | --- |
| *‎Negative Control* | - | AM4611 |
| *NFκB1* | Hs.618430 | s9504 |
| *AKIRIN2* | Hs.485915 | s30221 |
| *UBR5* | Hs.492445 | s224201 |
