# Supplementary material for: Hyd ubiquitinates the NF-κB co-factor Akirin to operate an effective immune response in Drosophila
Source: PLoS Pathog. 2020 Apr 27;16(4):e1008458. doi: 10.1371/journal.ppat.1008458 (PMC7205318; doi:10.1371/journal.ppat.1008458)
Supplement: S5 Table — (DOCX) [file ppat.1008458.s014.docx]

**Table S5. Oligonucleotides used for quantitative real-time PCR.**

For *Drosophila* S2 cells and adult flies

| **Gene** | **Forward** | **Reverse** |
| --- | --- | --- |
| *Attacin-A* | GGCCCATGCCAATTTATTCA | AGCAAAGACCTTGGCATCCA |
| *Attacin-D* | TTTATGGAGCGGTCAACGCCAATG | TGCAAATTGAGTCCTCCGCCAAAC |
| *Rp49* | GACGCTTCAAGGGACAGTATCTG | AAACGCGGTTCTGCATGAG |
| *Relish* | GGTGATAGTGCCCTGCATGT | CCATACCCAGCAAAGGTCGT |
| *Akirin* | CCGAACCTAGTCCGTTCAGTG | CTTGTGCAGTCTCTTGATCTCAT |
| *HyD* | GAGGTGGTTCTACAGGGCAAG | ATAAGGTCTTCGGGCACGTAA |
| *Bon* | AAAGGTCGGAGTCAAACTCTTCG | AAGGCATTCTAACAGCTTGGG |
| *Diap2* | CTCTTGTCCCGATCTCTTGTTG | GGTAGTAGAAACCTGCCTTTGC |
| *M-cup* | ACAAAGCTCAGTCACGACCTG | GACGAGAATCGCGGGGTAG |
| *Mkrn1* | AGACCATCTGCCGCTACTAC | TGCTGCTTGTACTAGGCTTCG |
| *Mura* | ACTTGAACAACCCGTCCTCAT | GTTCGGAGTTTCCAAAGTGGTTA |

For mammalian HeLa cells

| **Gene** | **Reference** (PrimePCR™ SYBR® Green Assay BioRad) |
| --- | --- |
| *IL6* | qHsaCID0020314 |
| *IL8* | qHsaCED0046633 |
| *GAPDH* | qHsaCED0038674 |
| *NFκB1* | qHsaCED0002379 |
| *AKIRIN2* | qHsaCID0011447 |
| *UBR5* | qHsaCID0014740 |
