# Supplementary figures and images for: Correction: Hyd ubiquitinates the NF-κB co-factor Akirin to operate an effective immune response in Drosophila
Source: PLoS Pathog. 2025 Dec 18;21(12):e1013785. doi: 10.1371/journal.ppat.1013785 (PMC12714183; doi:10.1371/journal.ppat.1013785)

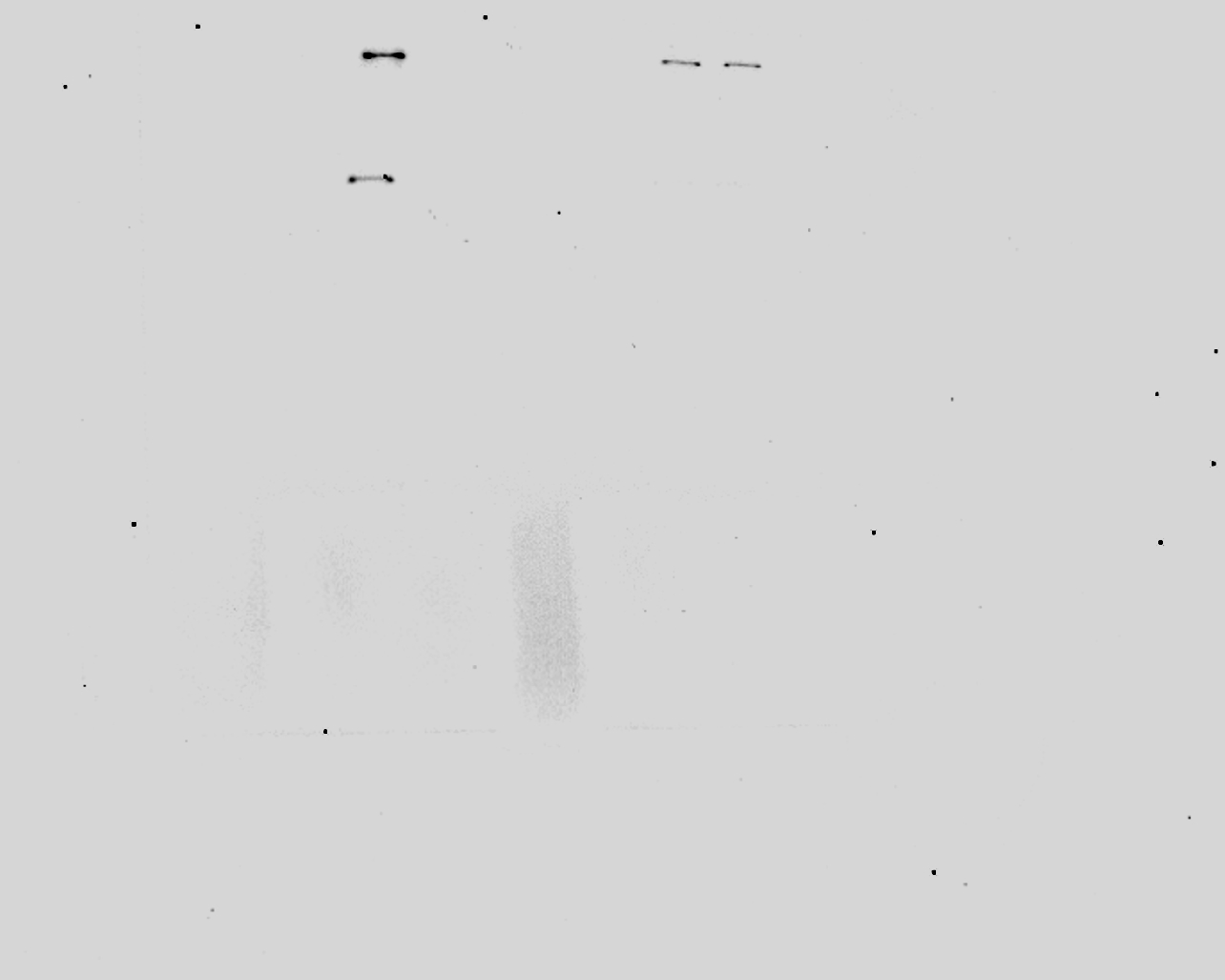

Supplement: S1 File — (ZIP) [file ppat.1013785.s001.zip › S1 File - Main Figure Data/Fig3/3A/Ak-HyD-2.0_exp_09(Chemiluminescence).jpg]

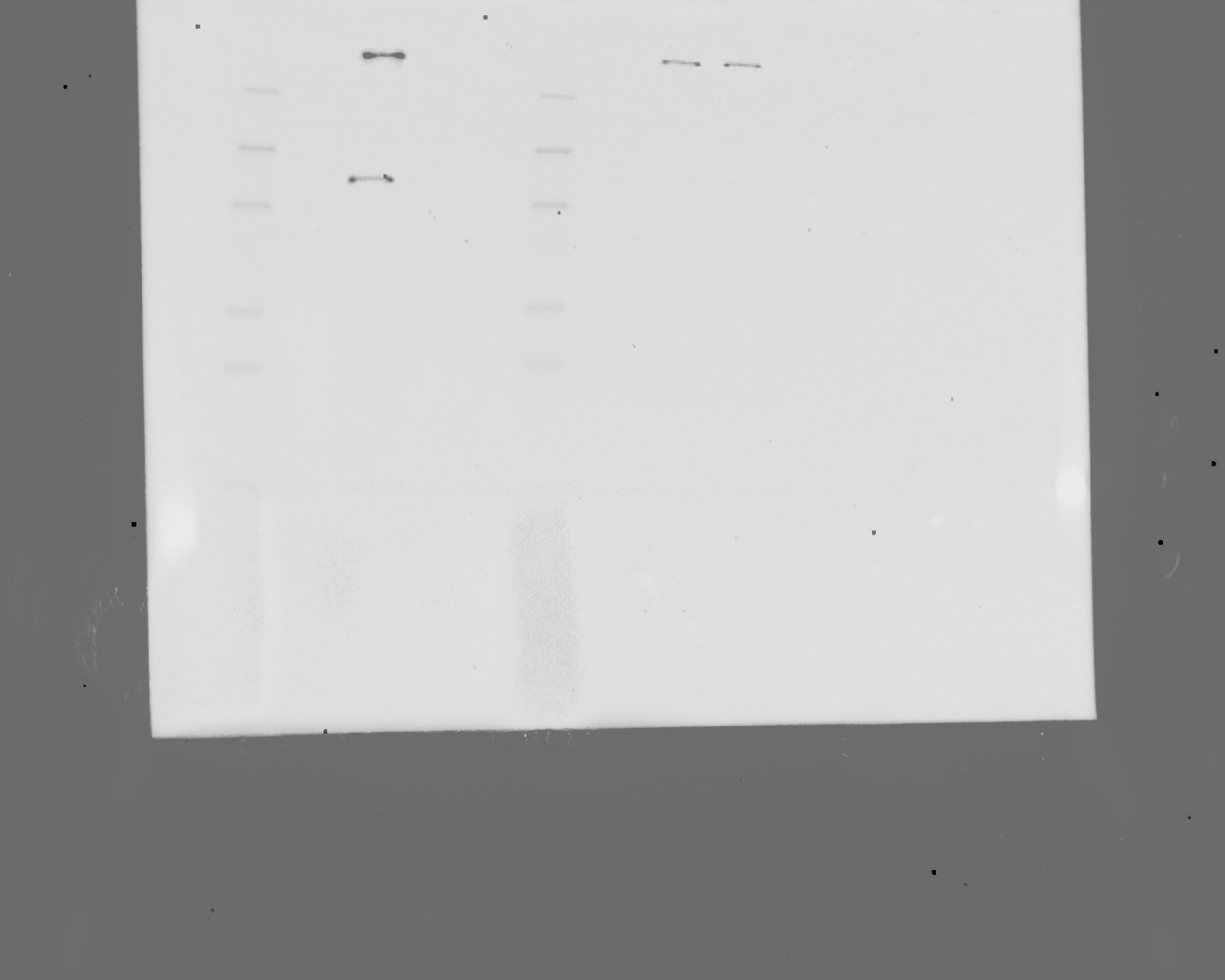

Supplement: S1 File — (ZIP) [file ppat.1013785.s001.zip › S1 File - Main Figure Data/Fig3/3A/Ak-HyD-2.0_exp_09(Composite).jpg]

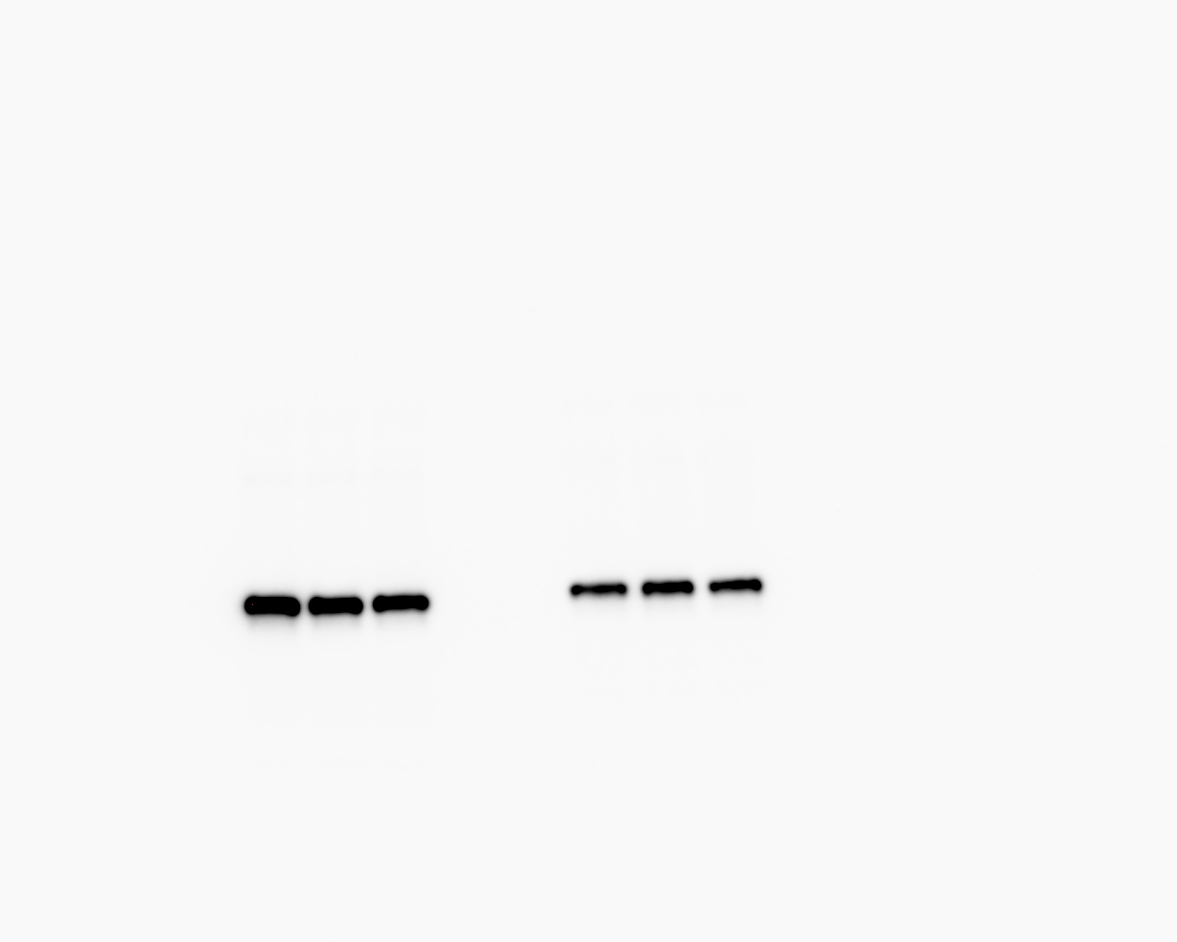

Supplement: S1 File — (ZIP) [file ppat.1013785.s001.zip › S1 File - Main Figure Data/Fig3/3A/Ak-HyD_Ak-ctrl_02(Chemiluminescence).jpg]

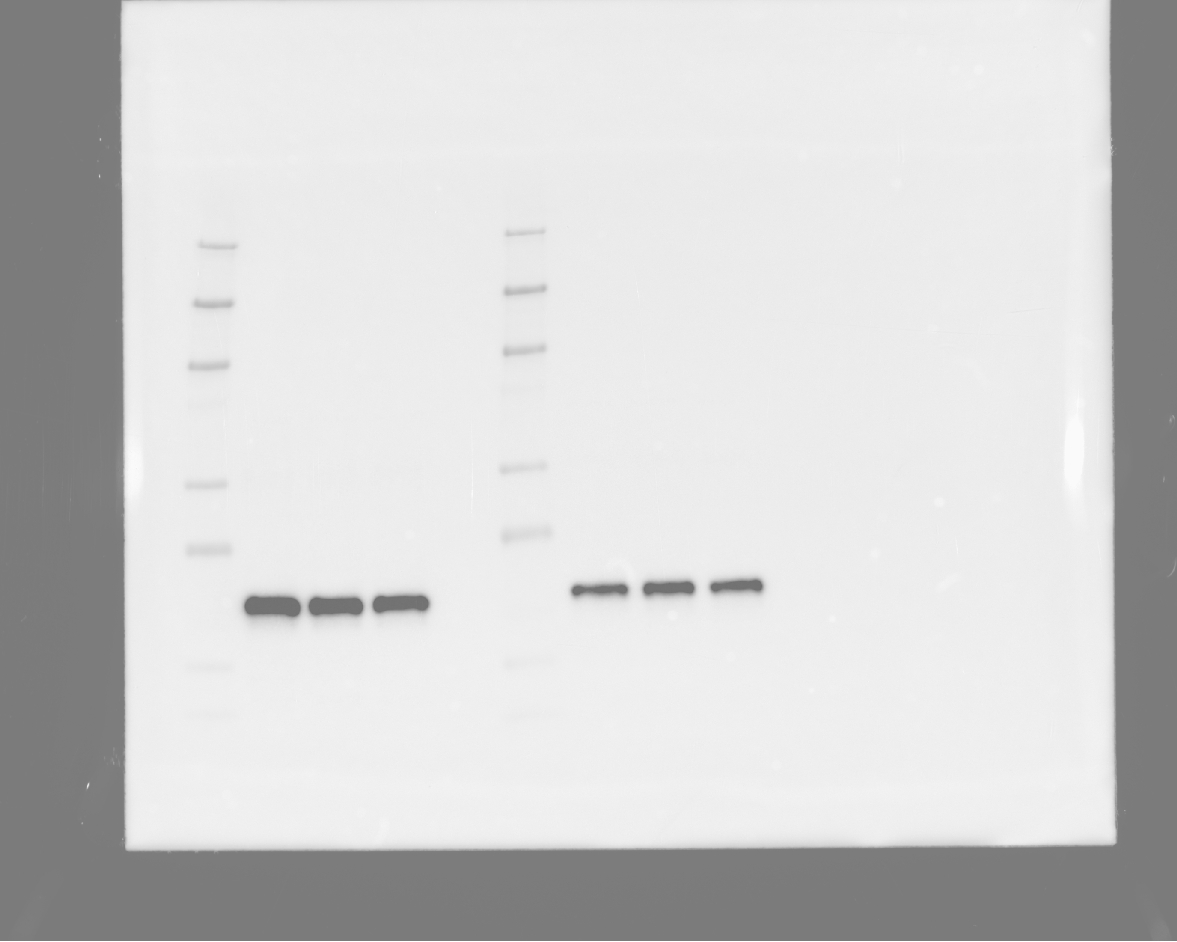

Supplement: S1 File — (ZIP) [file ppat.1013785.s001.zip › S1 File - Main Figure Data/Fig3/3A/Ak-HyD_Ak-ctrl_16(Composite).jpg]

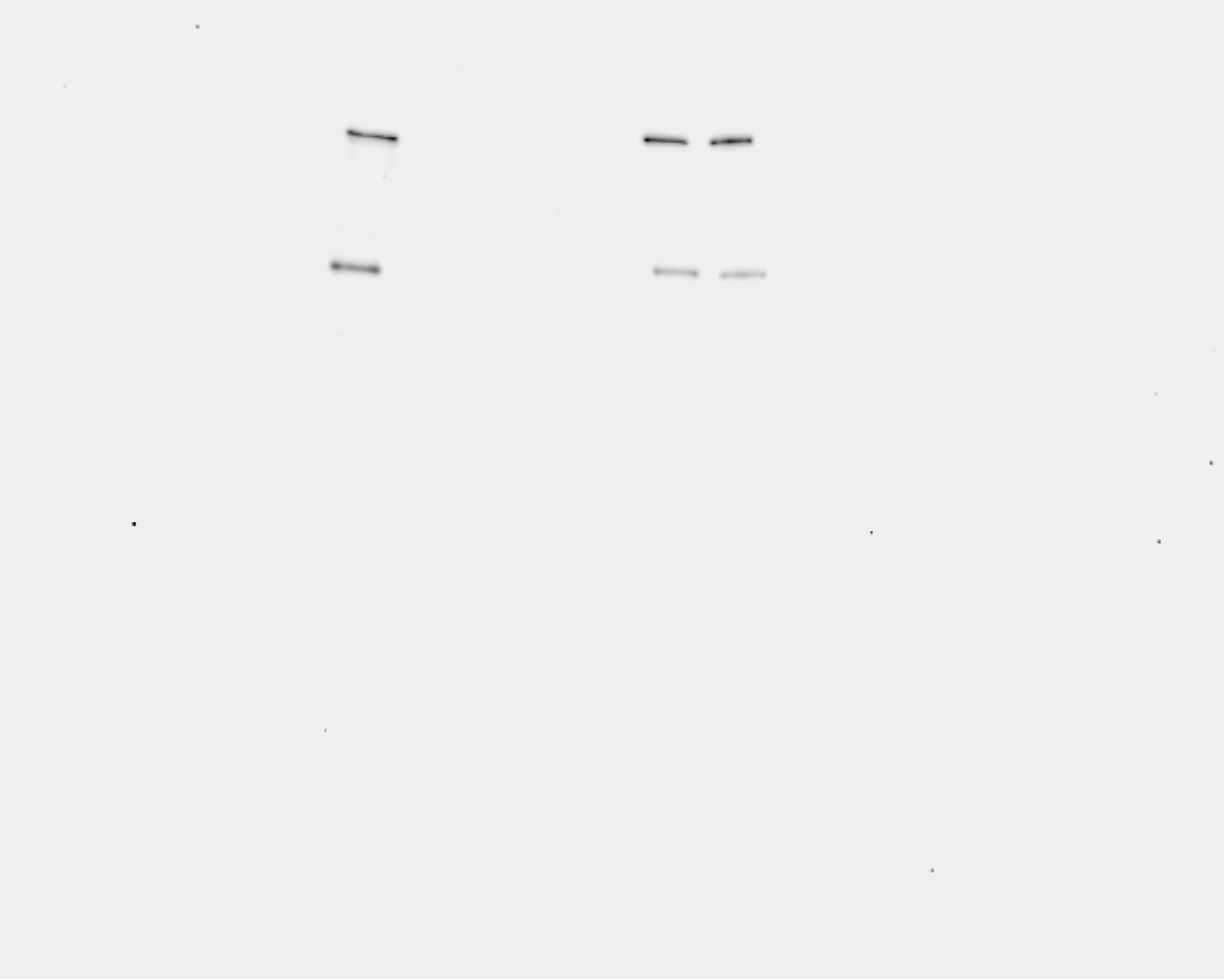

Supplement: S1 File — (ZIP) [file ppat.1013785.s001.zip › S1 File - Main Figure Data/Fig3/3A/Ak-HyD_exp_10(Chemiluminescence).jpg]

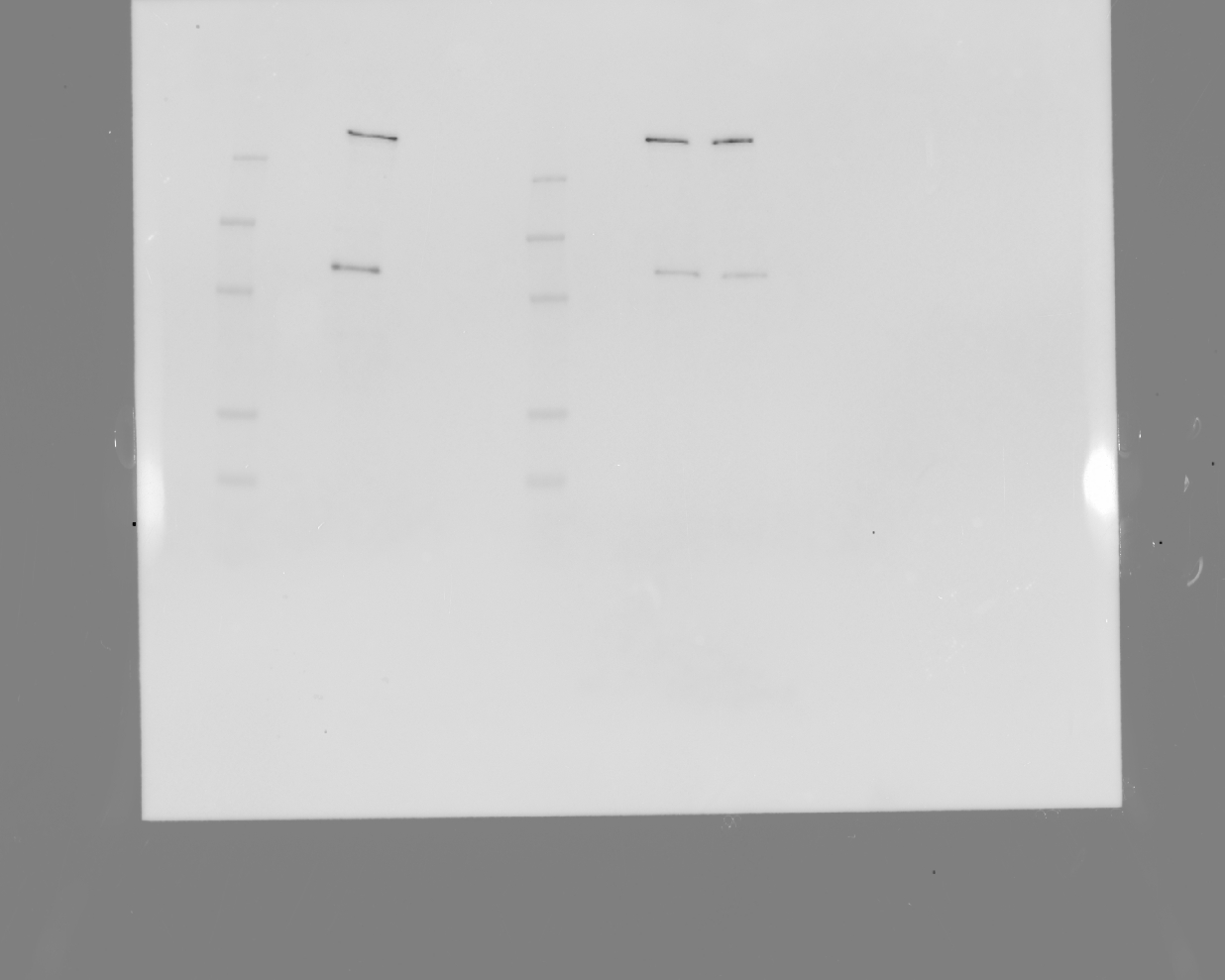

Supplement: S1 File — (ZIP) [file ppat.1013785.s001.zip › S1 File - Main Figure Data/Fig3/3A/Ak-HyD_exp_23(Composite).jpg]

## Slide 1
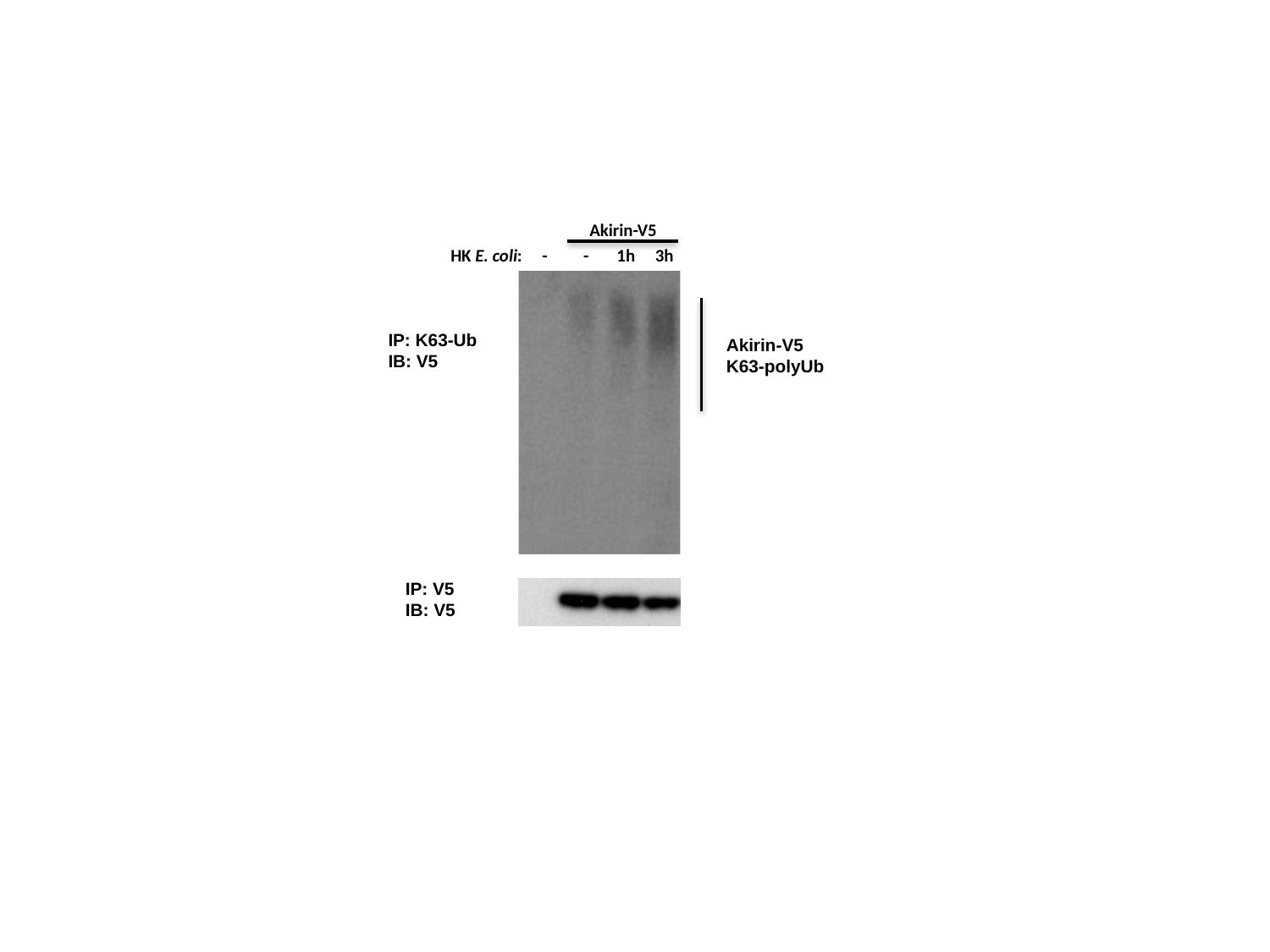

Akirin-V5
HK E. coli: - - 1h 3h
IP: K63-Ub
IB: V5
Akirin-V5
K63-polyUb
IP: V5
IB: V5

Supplement: S1 File — (ZIP) [file ppat.1013785.s001.zip › S1 File - Main Figure Data/Fig3/3B/3-B.pptx]

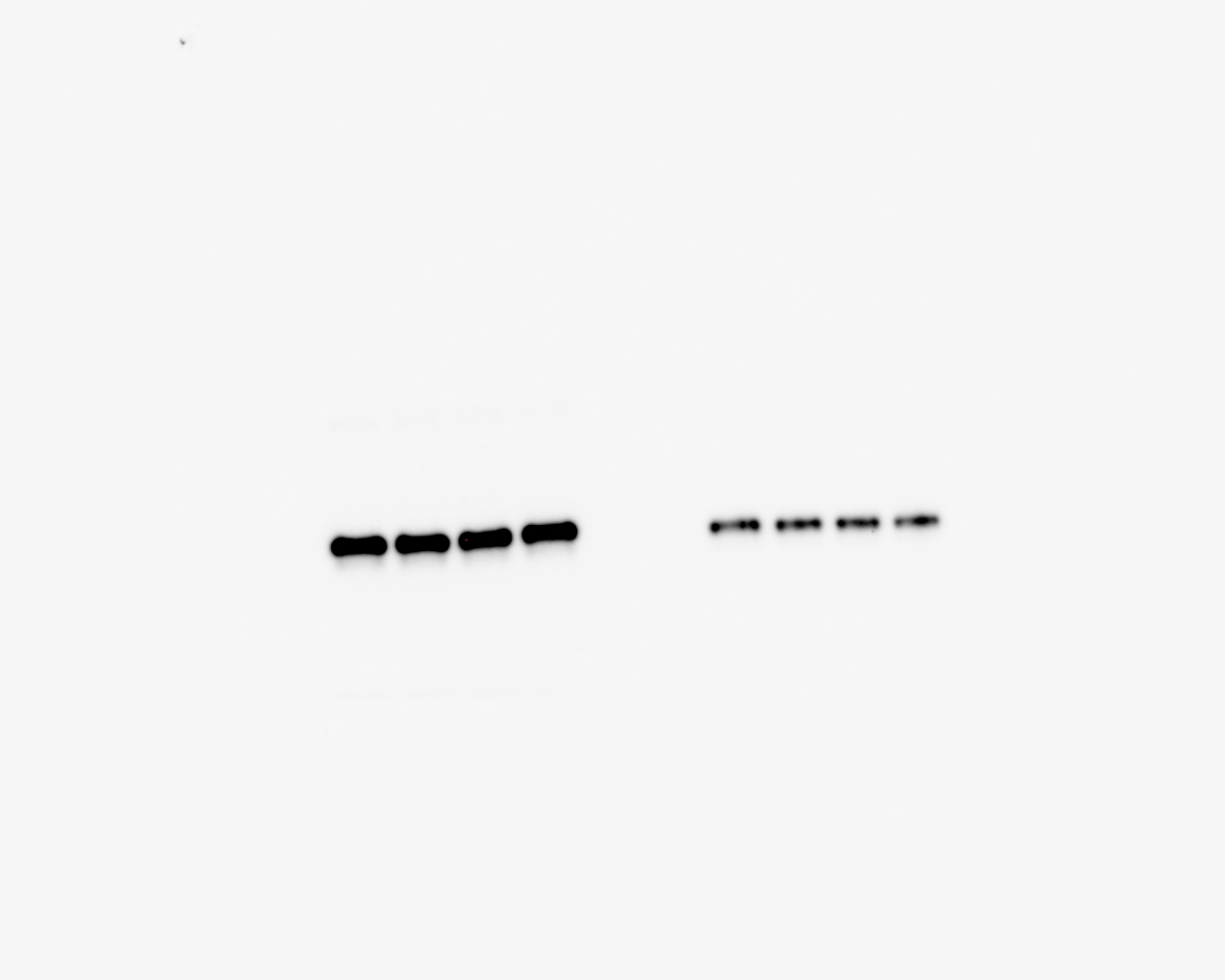

Supplement: S1 File — (ZIP) [file ppat.1013785.s001.zip › S1 File - Main Figure Data/Fig3/3C/Ak_ub-HyD_Kd_Ak-ctrl_02(Chemiluminescence).jpg]

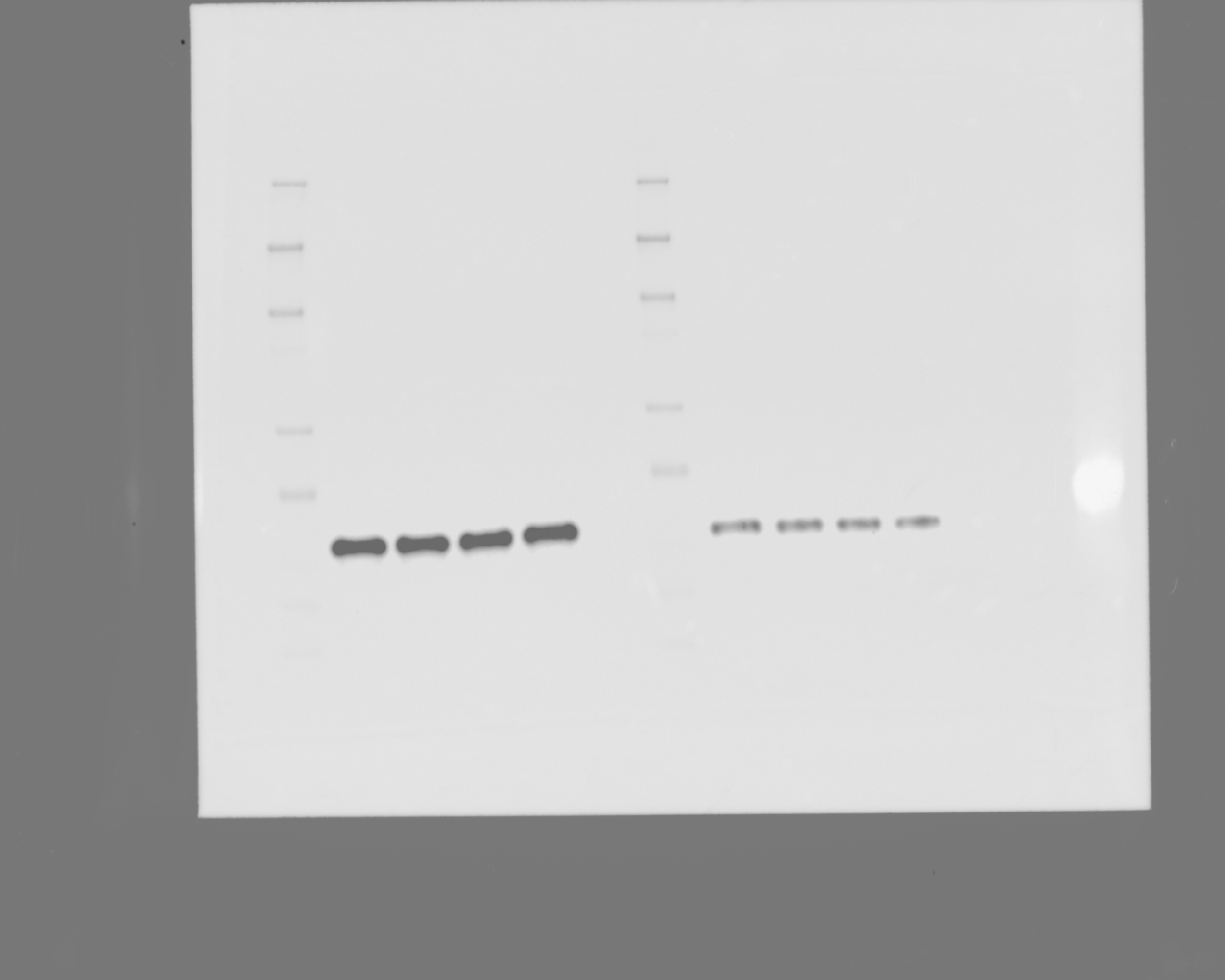

Supplement: S1 File — (ZIP) [file ppat.1013785.s001.zip › S1 File - Main Figure Data/Fig3/3C/Ak_ub-HyD_Kd_Ak-ctrl_24(Composite).jpg]

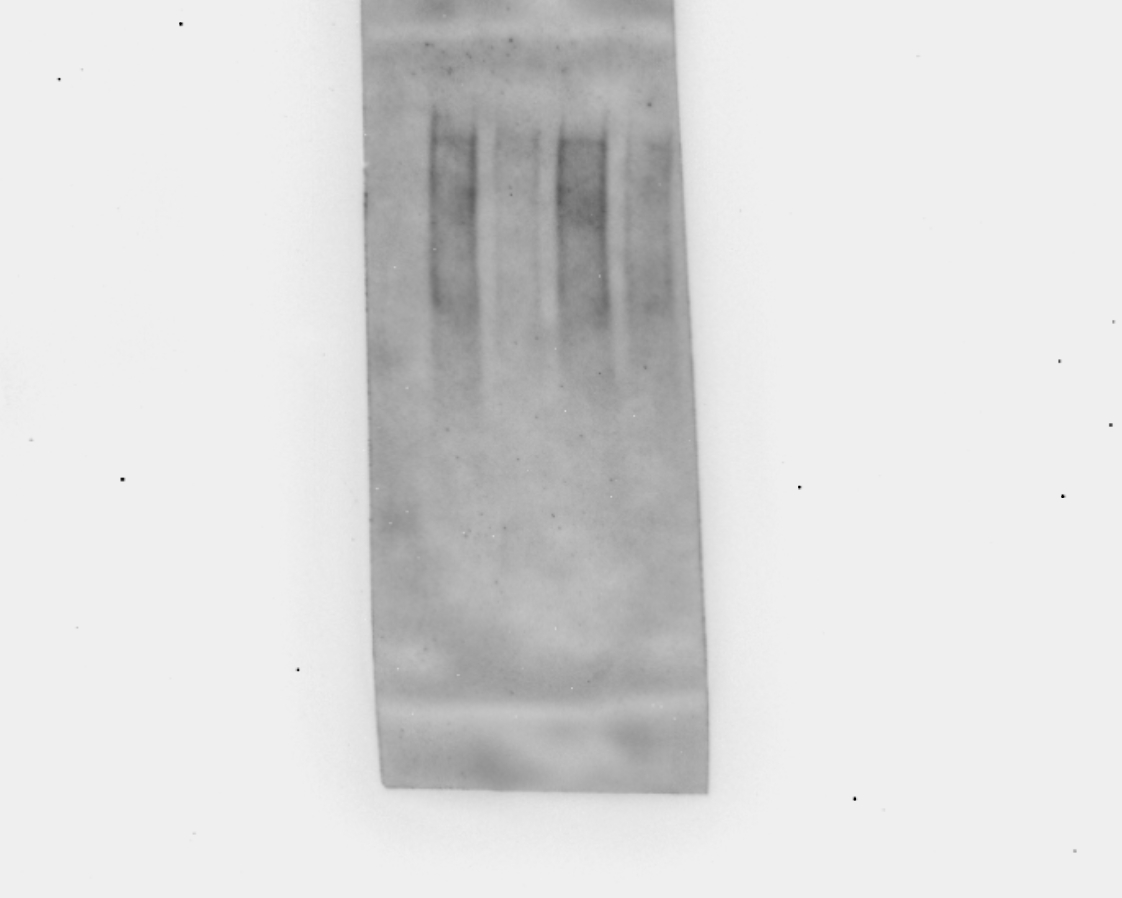

Supplement: S1 File — (ZIP) [file ppat.1013785.s001.zip › S1 File - Main Figure Data/Fig3/3C/Alex 2017-12-14 20h06m22s(Chemiluminescence).jpg]

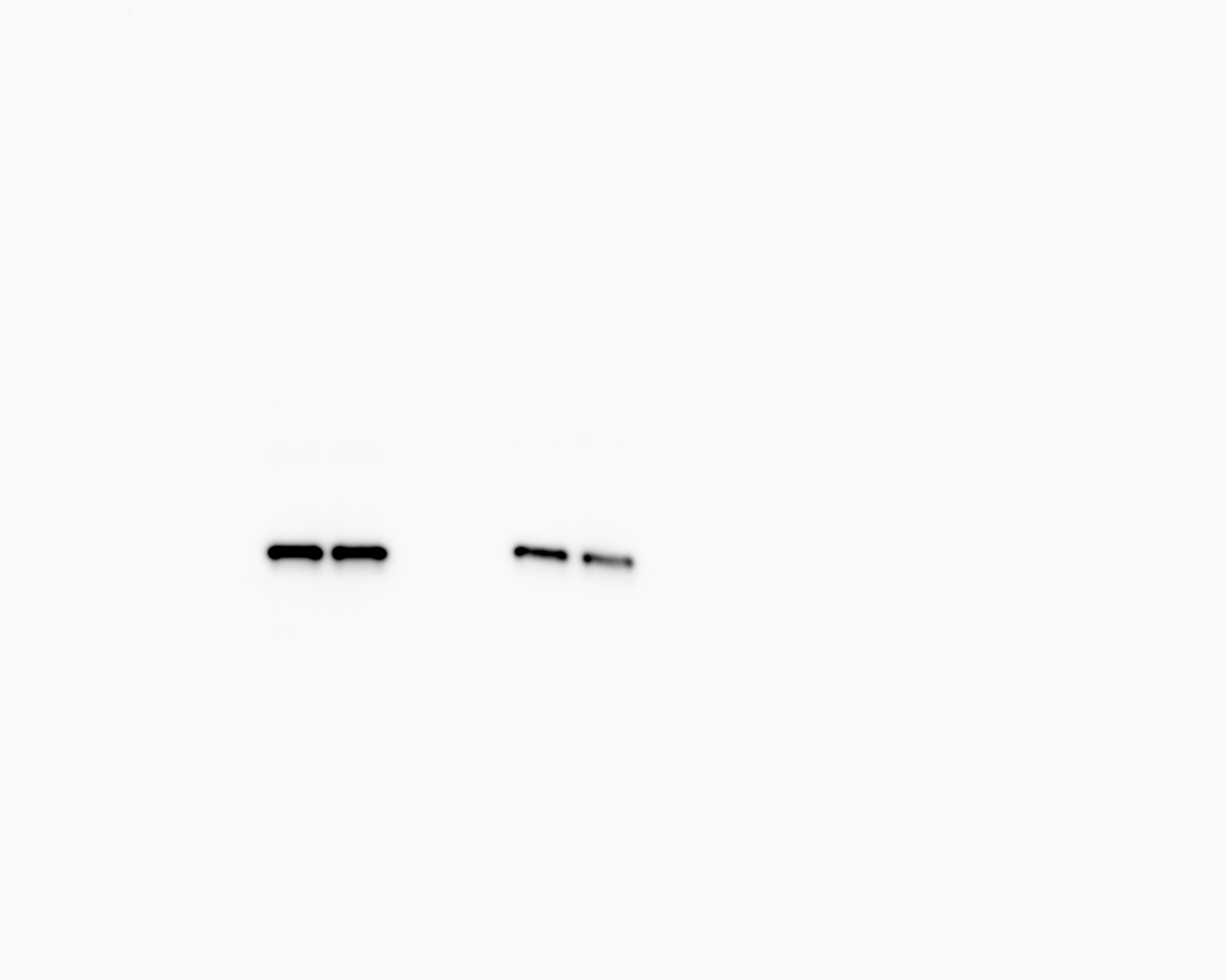

Supplement: S1 File — (ZIP) [file ppat.1013785.s001.zip › S1 File - Main Figure Data/Fig3/3D/Ak-Bap_Ak_ctrl_06(Chemiluminescence).jpg]

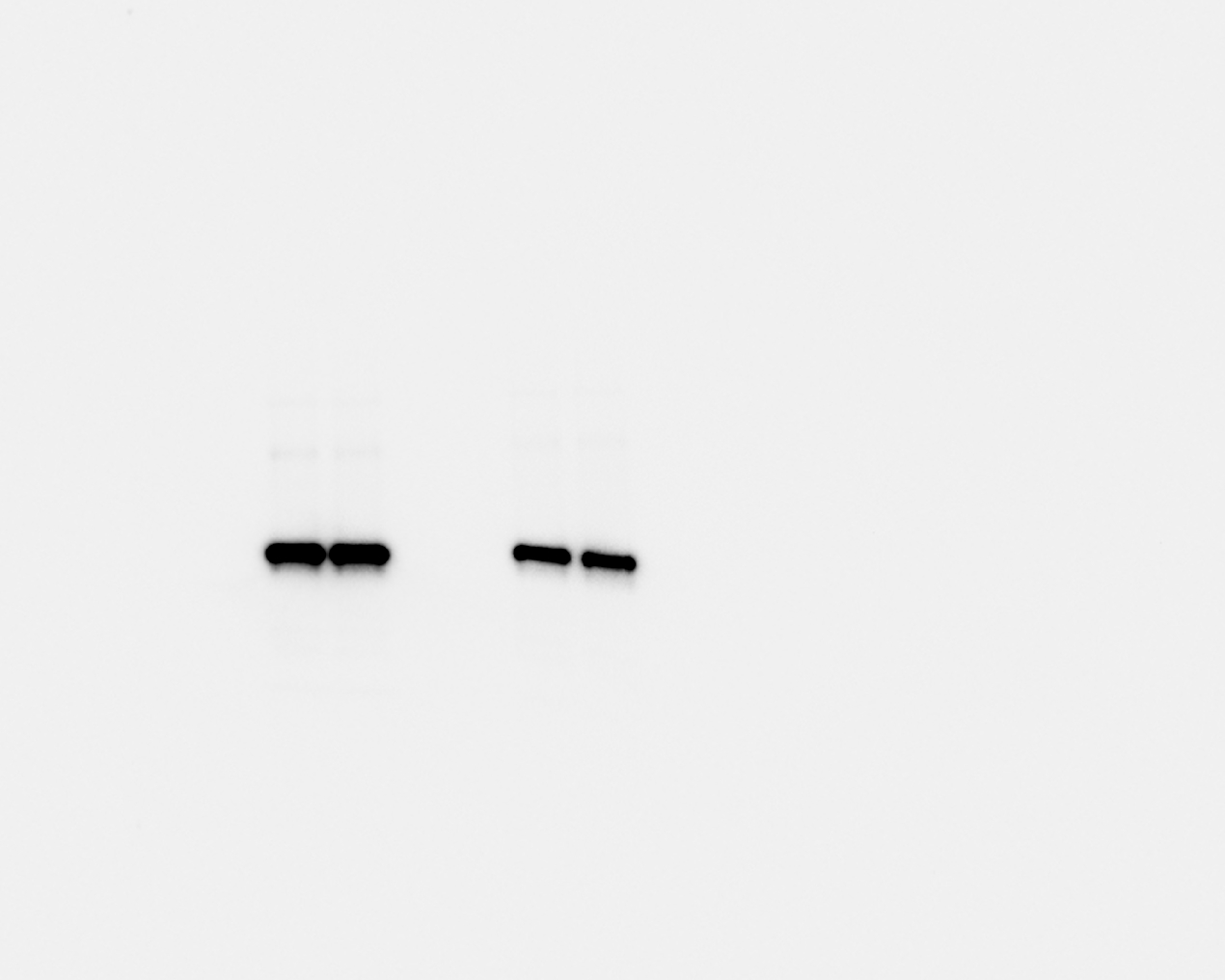

Supplement: S1 File — (ZIP) [file ppat.1013785.s001.zip › S1 File - Main Figure Data/Fig3/3D/Ak-Bap_Ak_ctrl_11(Chemiluminescence).jpg]

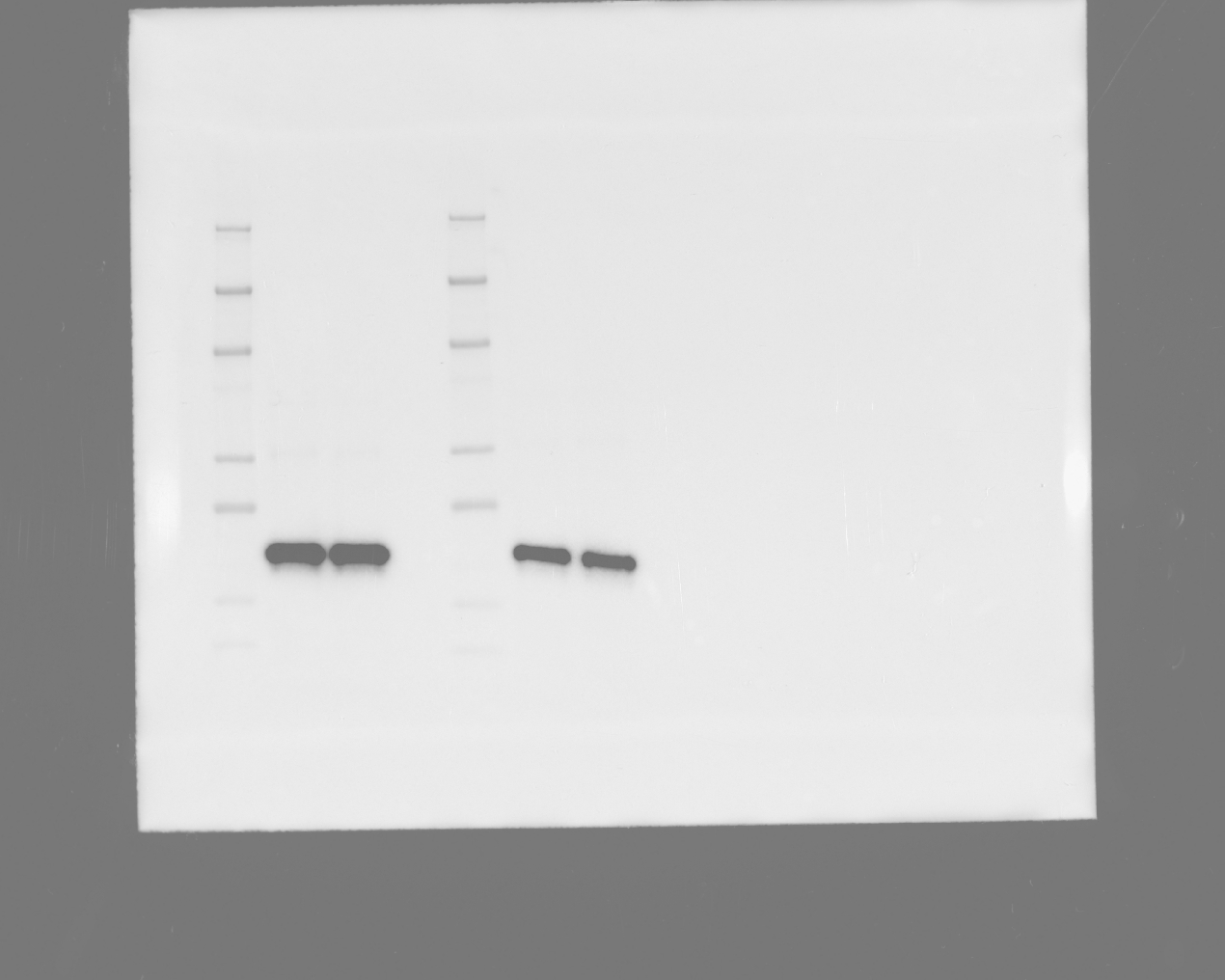

Supplement: S1 File — (ZIP) [file ppat.1013785.s001.zip › S1 File - Main Figure Data/Fig3/3D/Ak-Bap_Ak_ctrl_11(Composite).jpg]

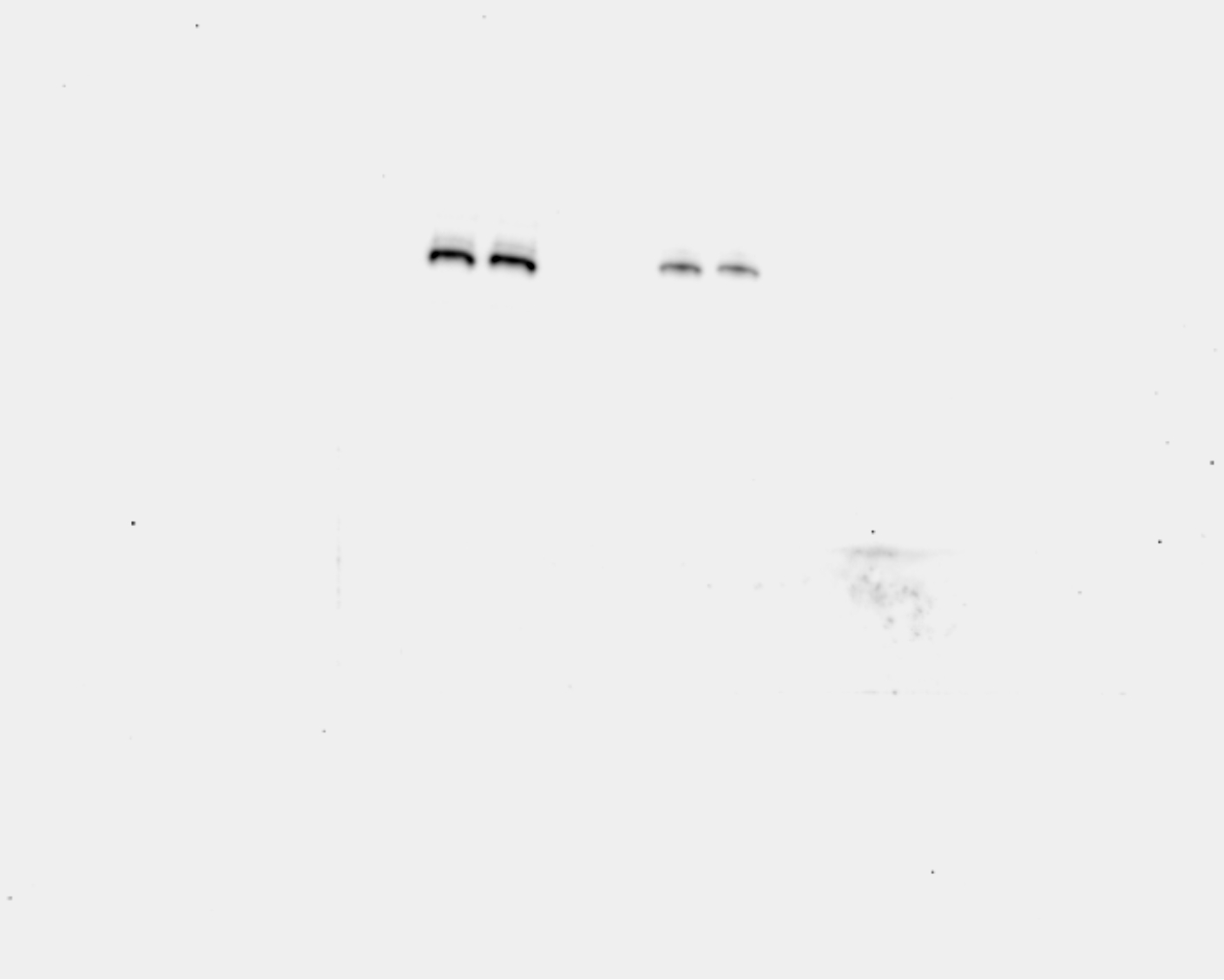

Supplement: S1 File — (ZIP) [file ppat.1013785.s001.zip › S1 File - Main Figure Data/Fig3/3D/Ak-Bap_exp_06(Chemiluminescence).jpg]

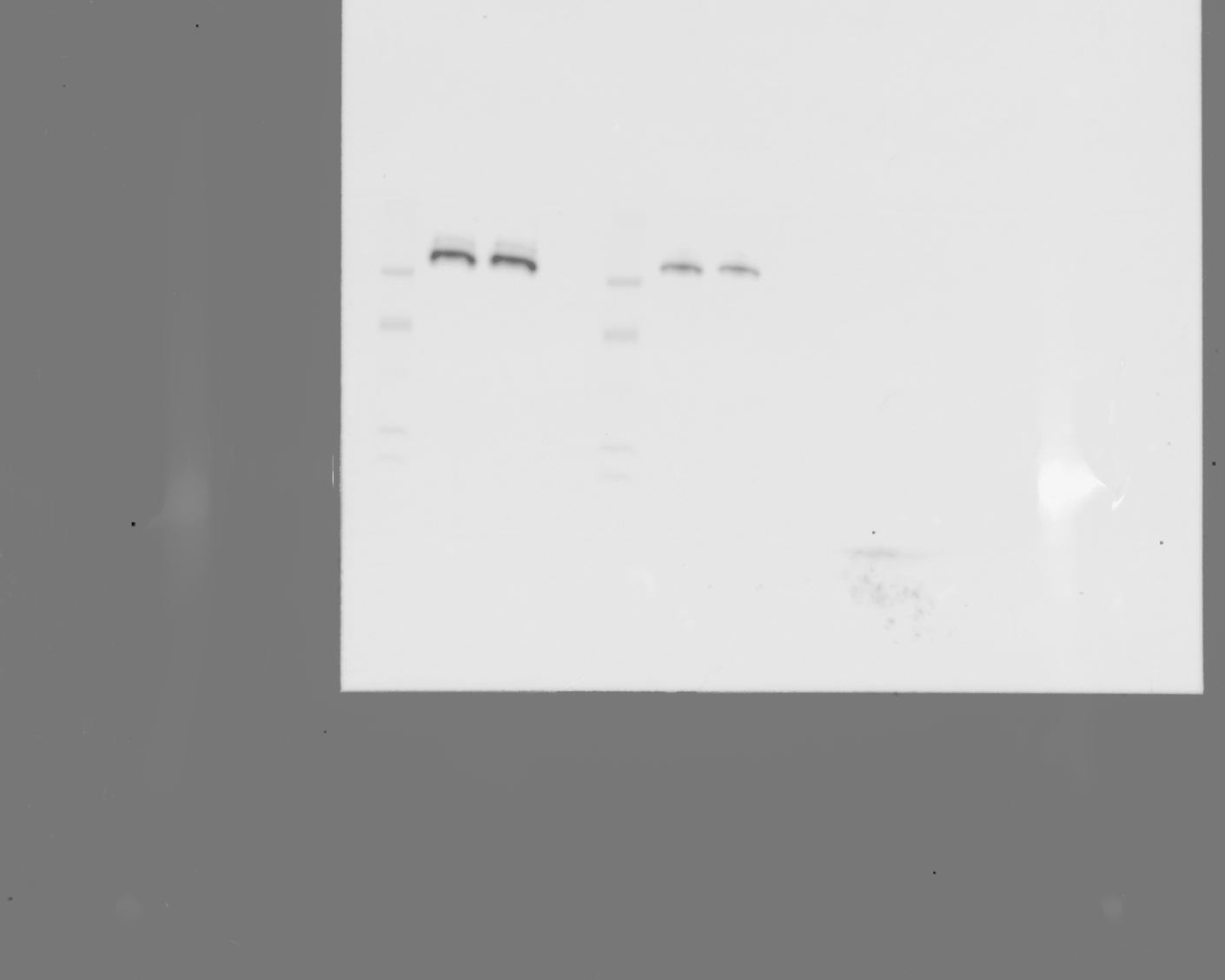

Supplement: S1 File — (ZIP) [file ppat.1013785.s001.zip › S1 File - Main Figure Data/Fig3/3D/Ak-Bap_exp_23(Composite).jpg]

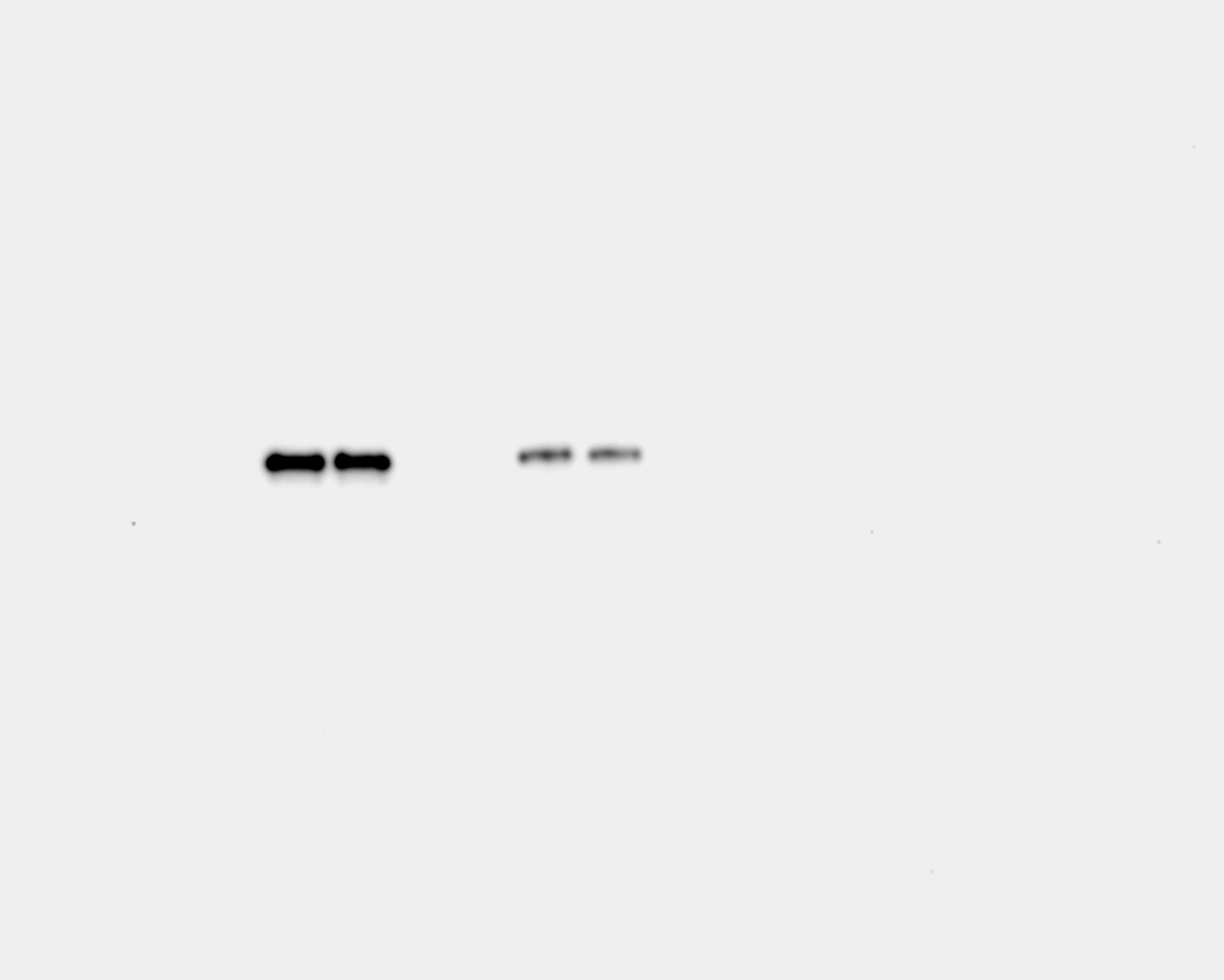

Supplement: S1 File — (ZIP) [file ppat.1013785.s001.zip › S1 File - Main Figure Data/Fig3/3D/Ak-Rel_Ak-ctrl_09(Chemiluminescence).jpg]

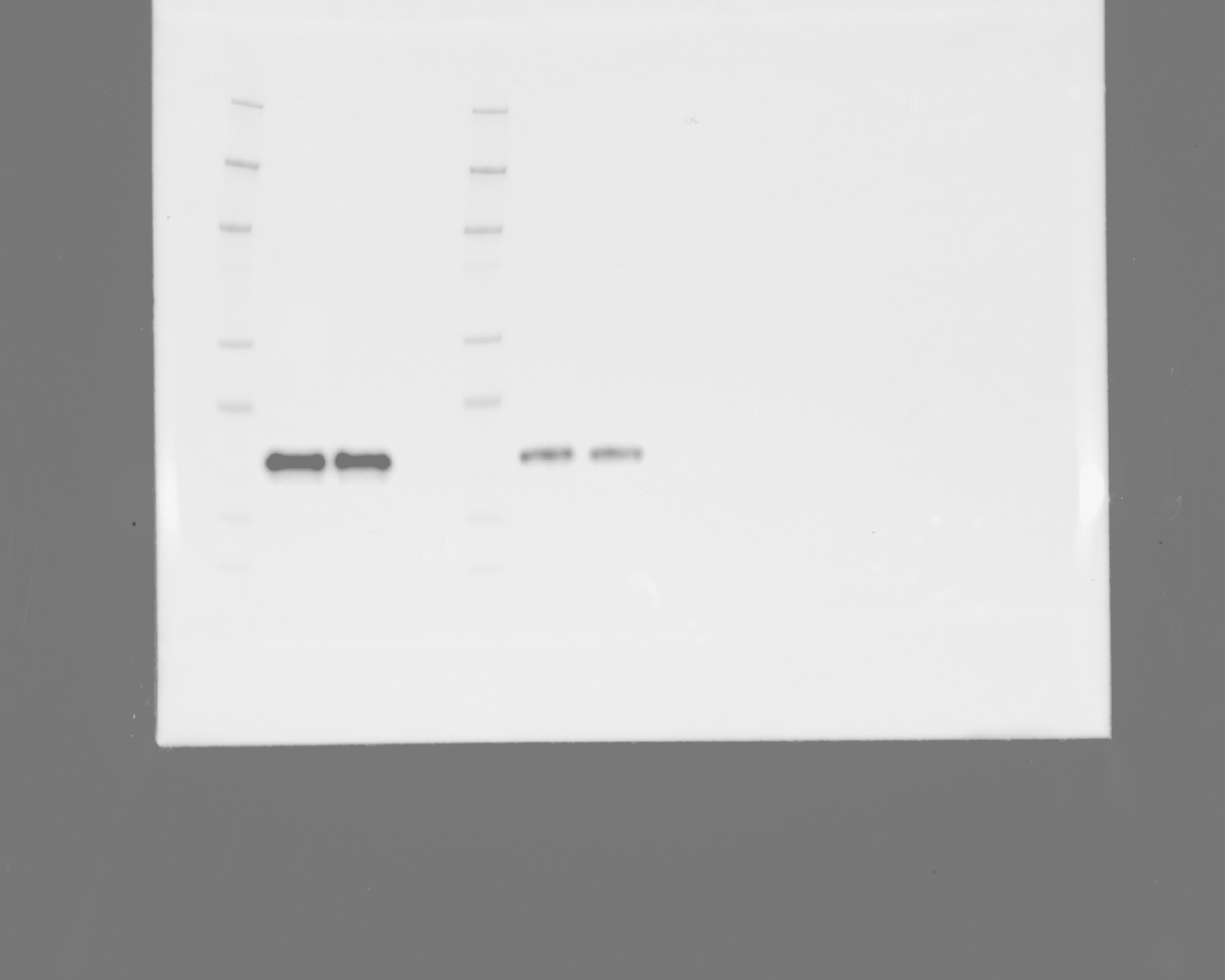

Supplement: S1 File — (ZIP) [file ppat.1013785.s001.zip › S1 File - Main Figure Data/Fig3/3D/Ak-Rel_Ak-ctrl_24(Composite).jpg]

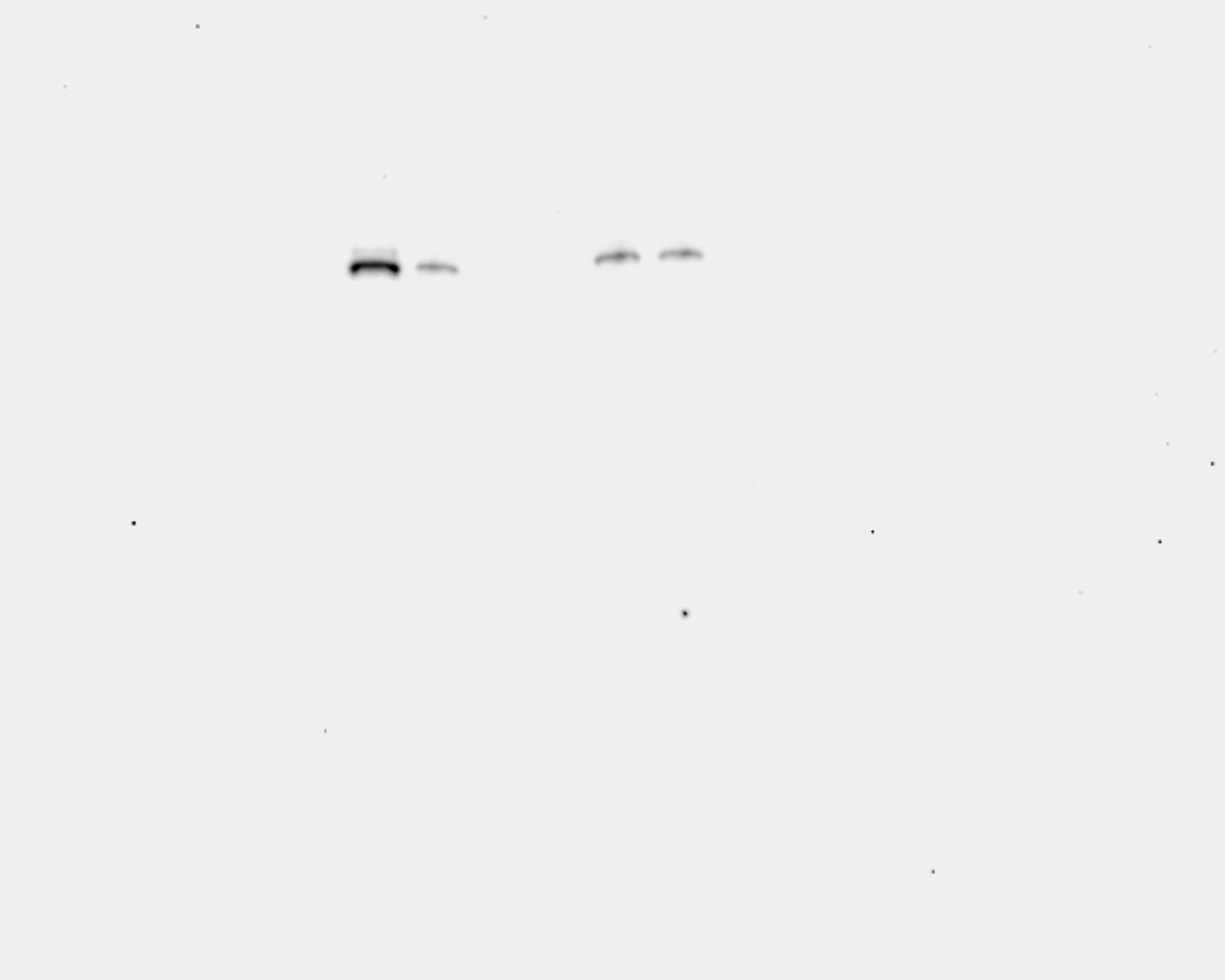

Supplement: S1 File — (ZIP) [file ppat.1013785.s001.zip › S1 File - Main Figure Data/Fig3/3D/Ak-Rel_exp_08(Chemiluminescence).jpg]

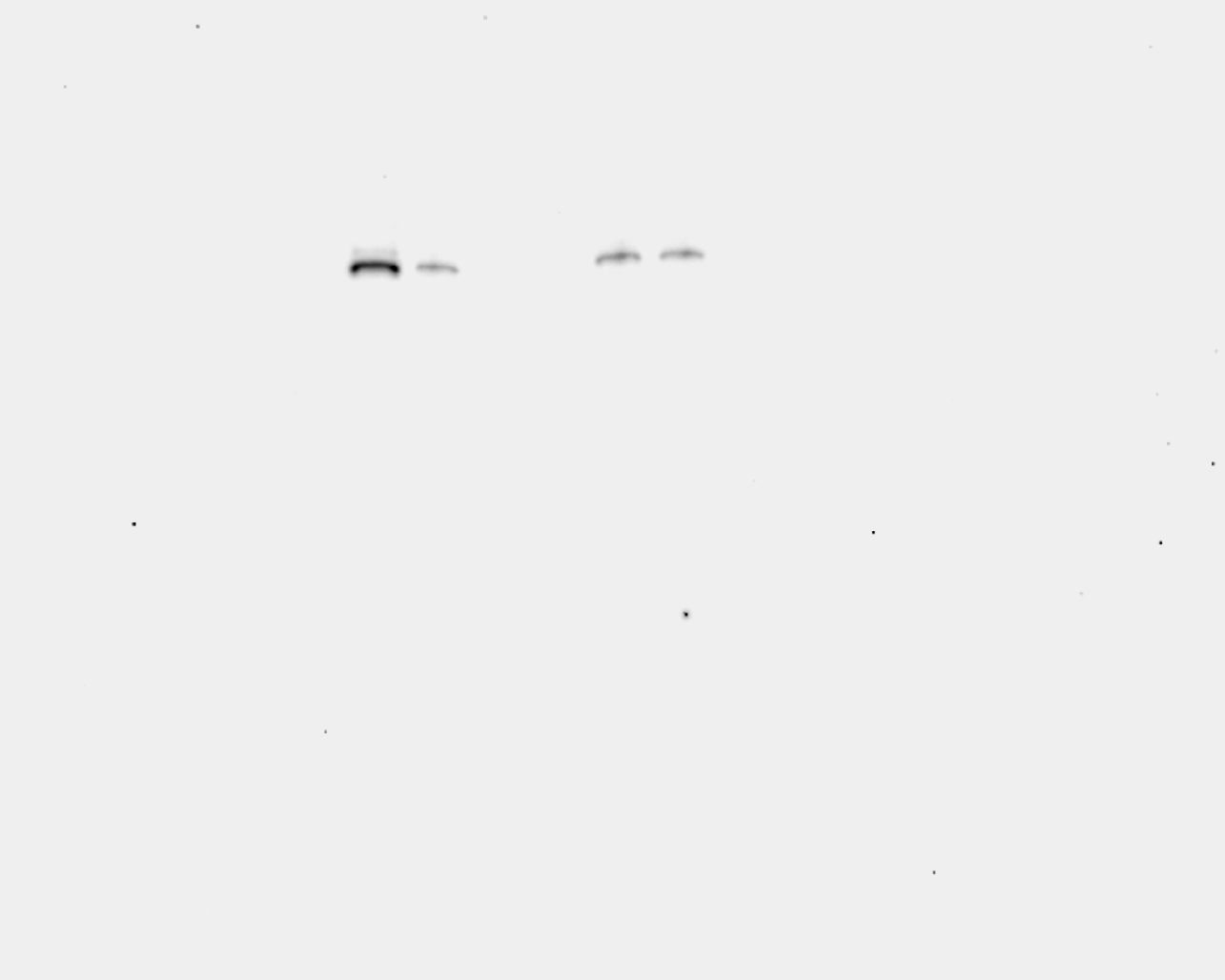

Supplement: S1 File — (ZIP) [file ppat.1013785.s001.zip › S1 File - Main Figure Data/Fig3/3D/Ak-Rel_exp_23(Chemiluminescence).jpg]

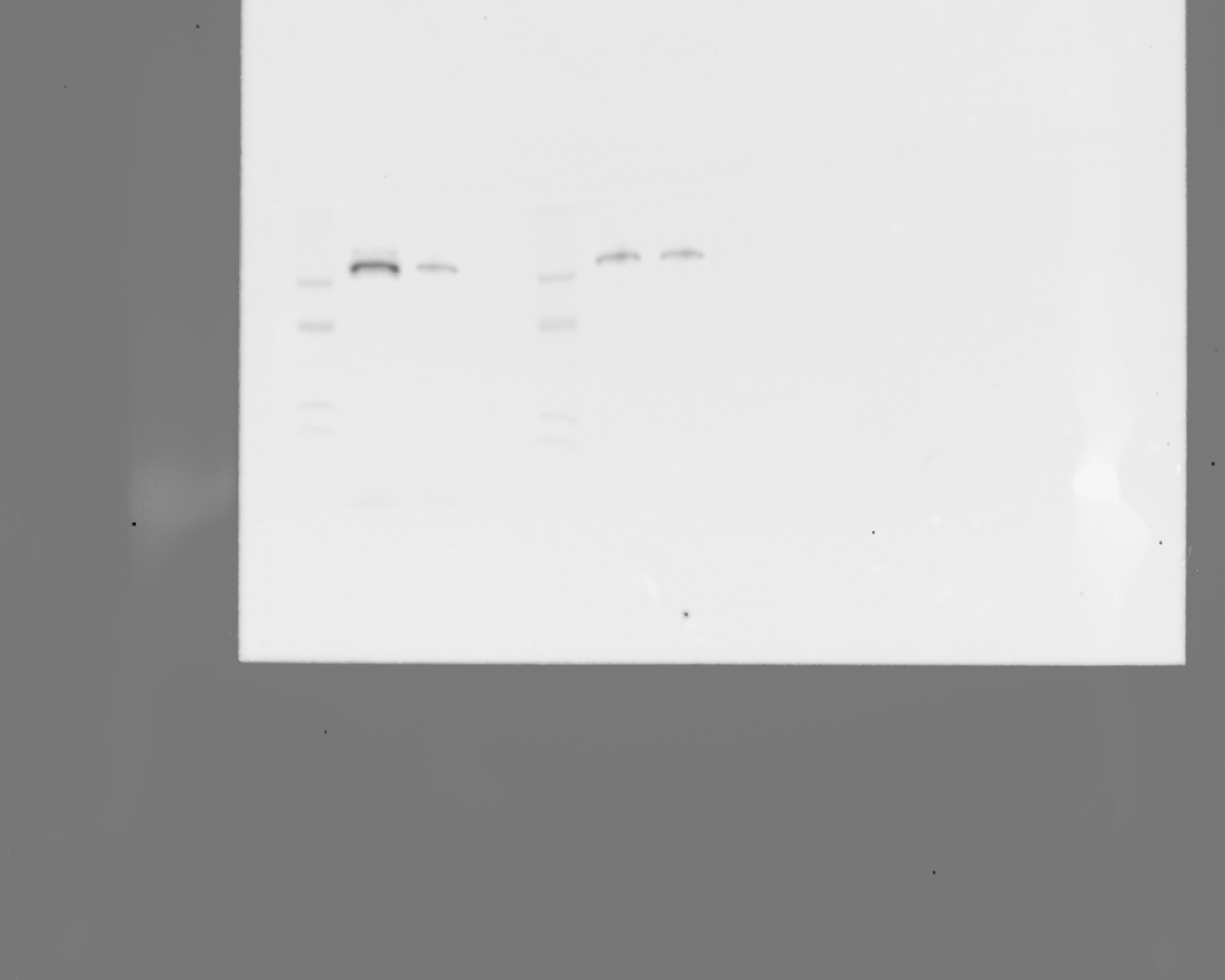

Supplement: S1 File — (ZIP) [file ppat.1013785.s001.zip › S1 File - Main Figure Data/Fig3/3D/Ak-Rel_exp_23(Composite).jpg]

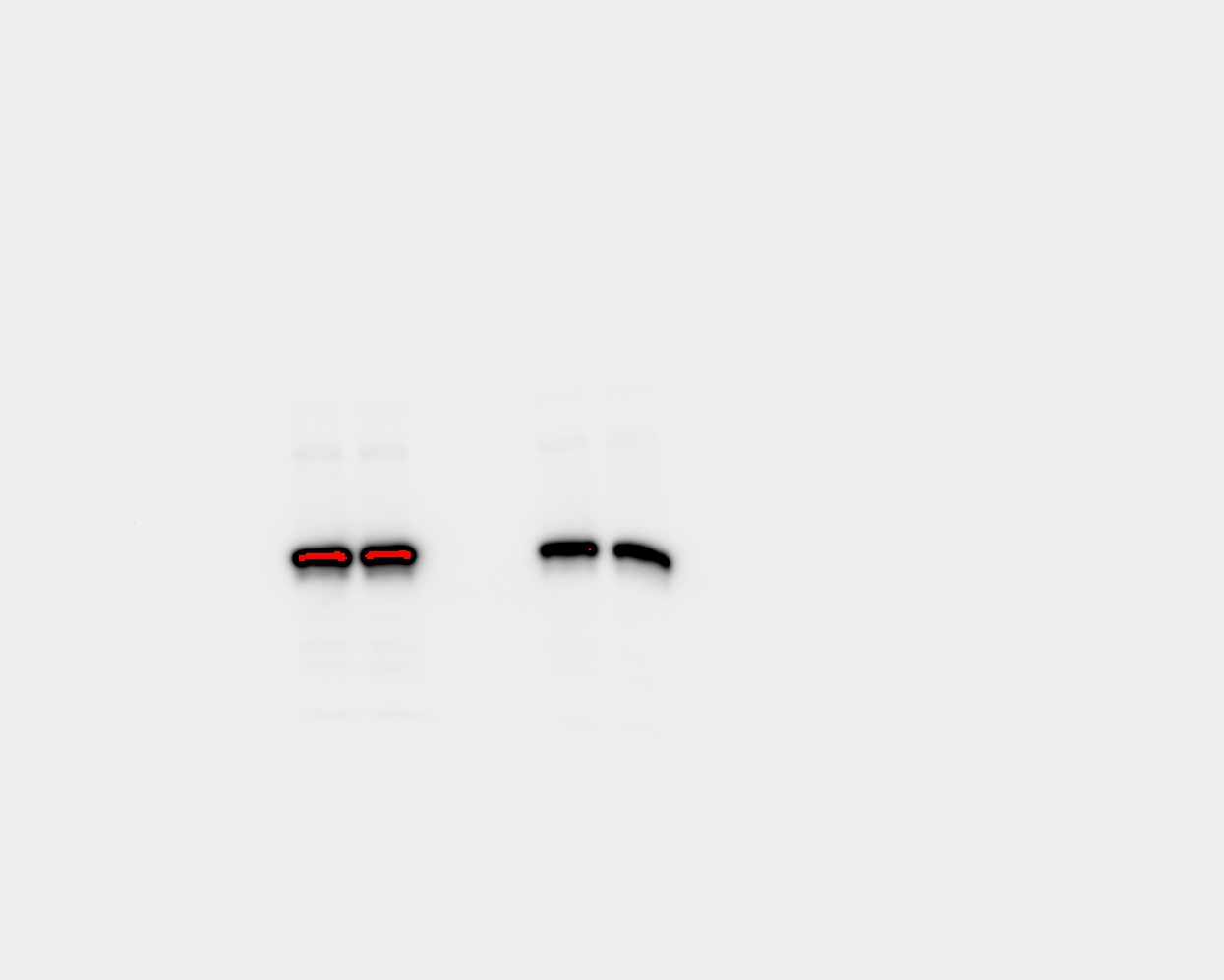

Supplement: S2 File — (ZIP) [file ppat.1013785.s002.zip › S2 File - Supplementary File Data/FigS3/Ak-Iap2_Ak-ctrl_04(Chemiluminescence).jpg]

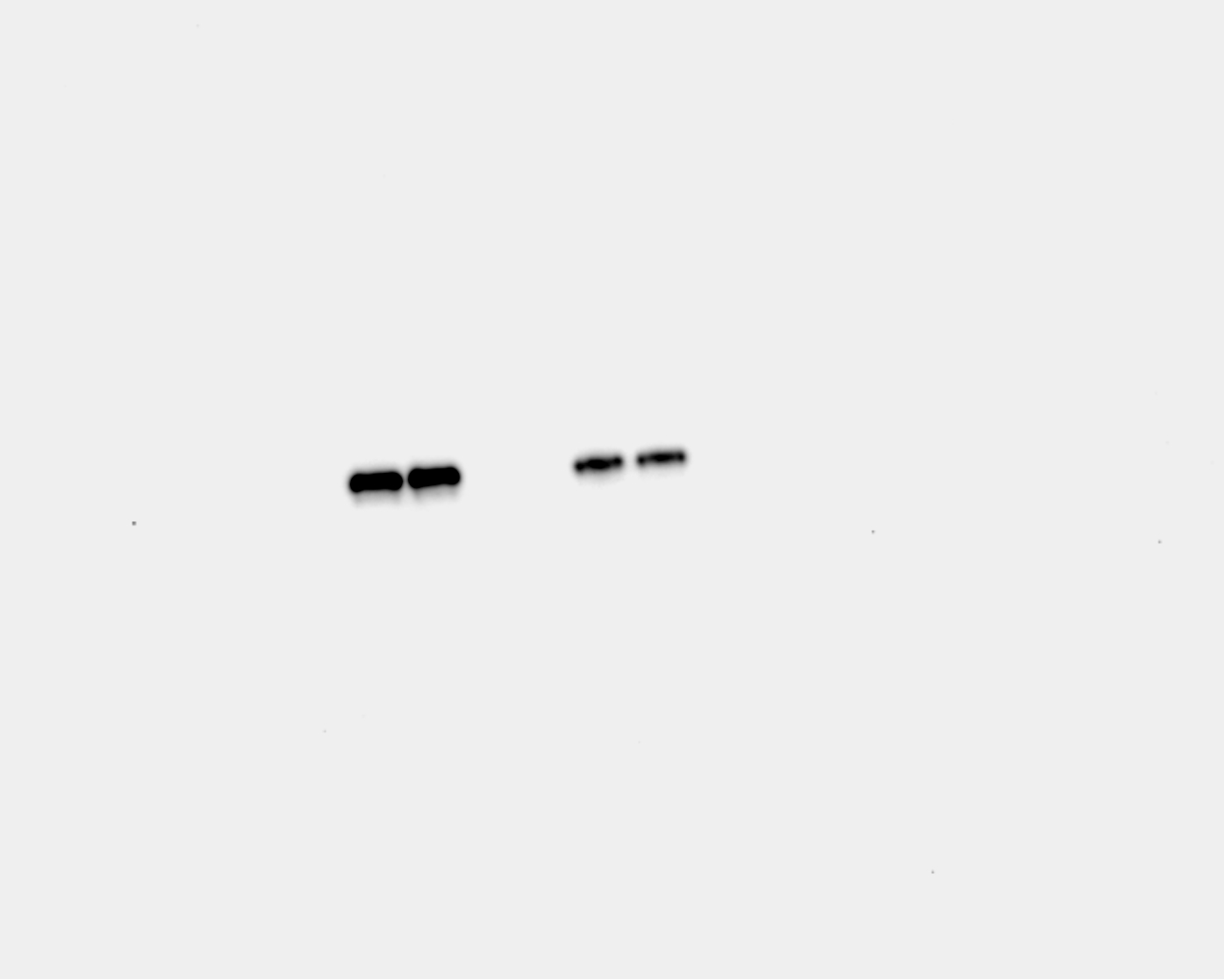

Supplement: S2 File — (ZIP) [file ppat.1013785.s002.zip › S2 File - Supplementary File Data/FigS3/Ak-IAP2_Ak-ctrl_22(Chemiluminescence).jpg]

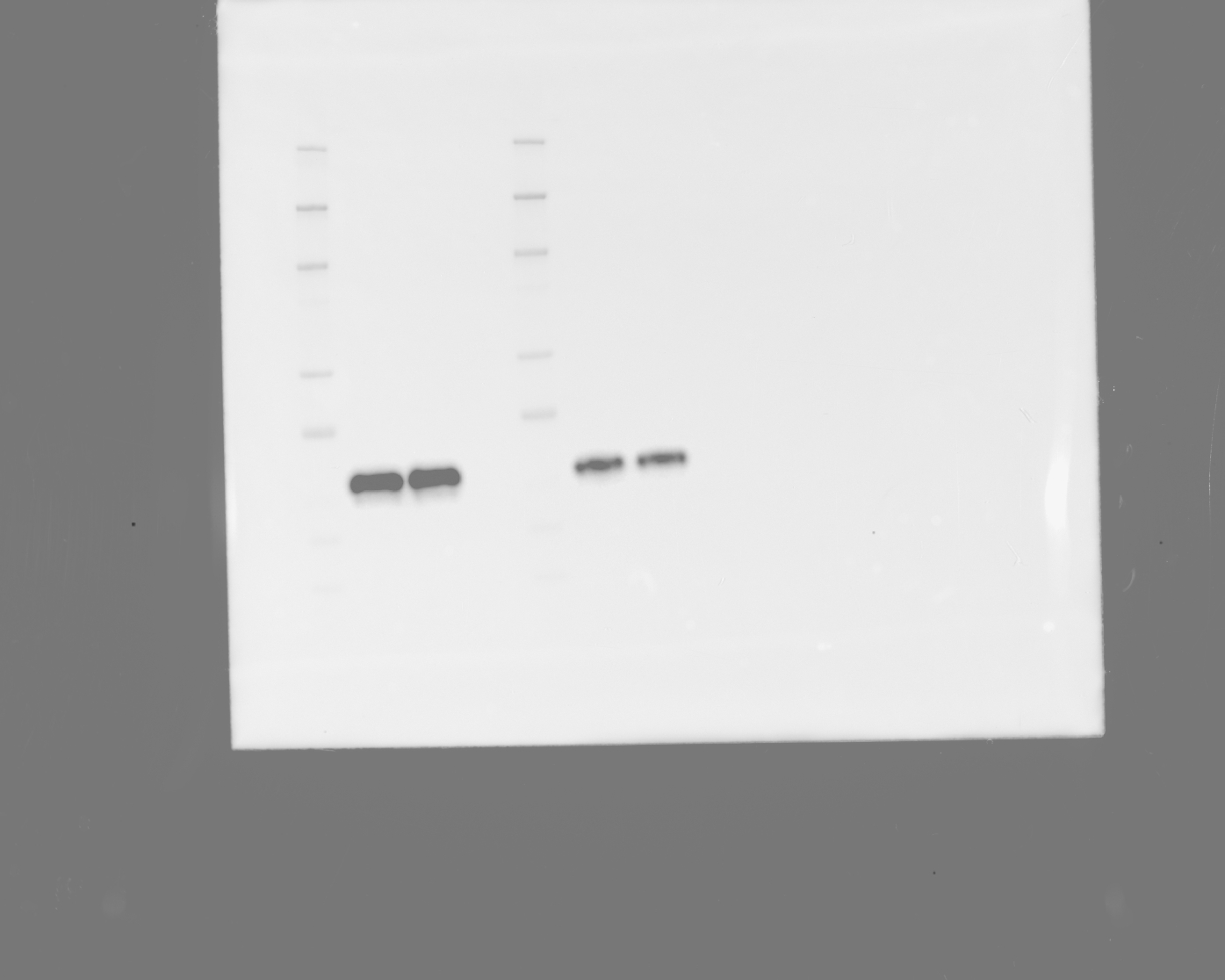

Supplement: S2 File — (ZIP) [file ppat.1013785.s002.zip › S2 File - Supplementary File Data/FigS3/Ak-IAP2_Ak-ctrl_30(Composite).jpg]

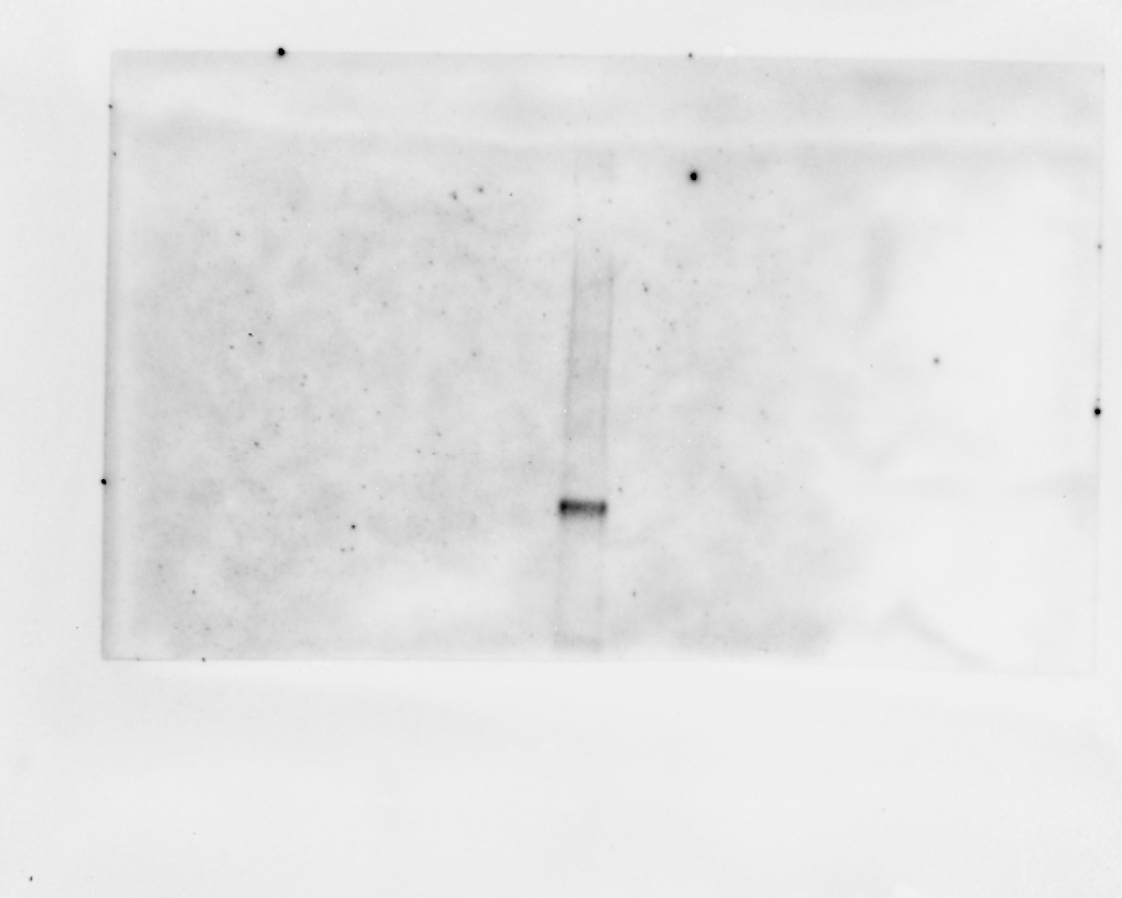

Supplement: S2 File — (ZIP) [file ppat.1013785.s002.zip › S2 File - Supplementary File Data/FigS3/Ak-Iap2_exp_03(Chemiluminescence).jpg]

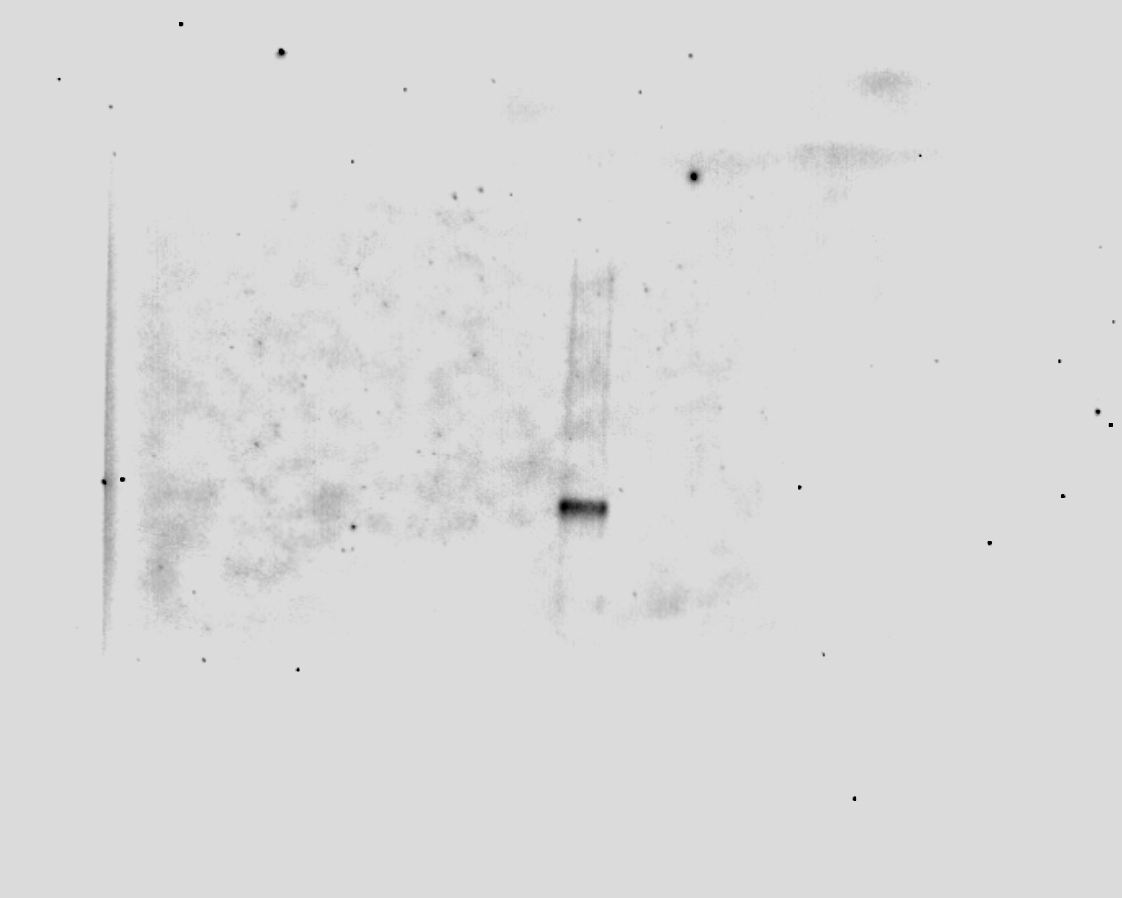

Supplement: S2 File — (ZIP) [file ppat.1013785.s002.zip › S2 File - Supplementary File Data/FigS3/Ak-Iap2_exp_24(Chemiluminescence).jpg]

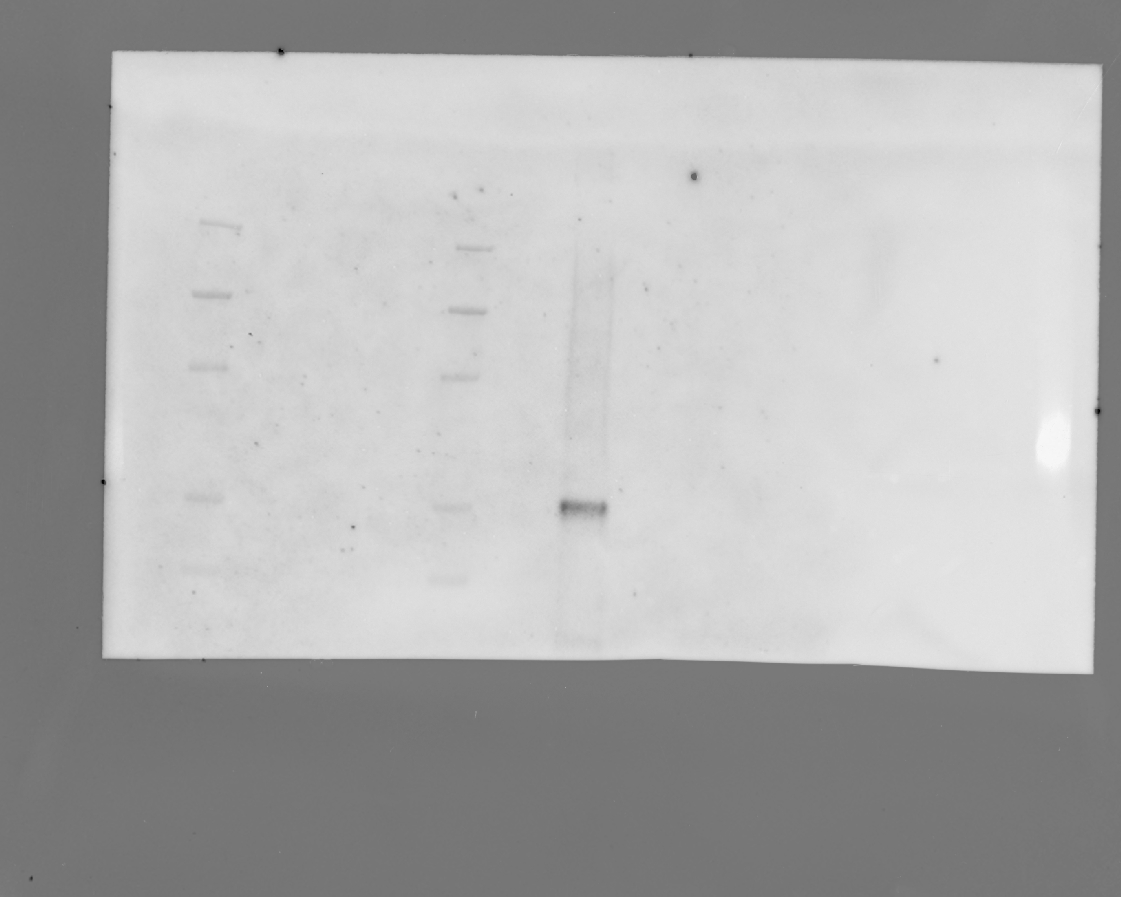

Supplement: S2 File — (ZIP) [file ppat.1013785.s002.zip › S2 File - Supplementary File Data/FigS3/Ak-Iap2_exp_25(Composite).jpg]
